# Gaseous Mediators as a Key Molecular Targets for the Development of Gastrointestinal-Safe Anti-Inflammatory Pharmacology

**DOI:** 10.3389/fphar.2021.657457

**Published:** 2021-04-29

**Authors:** Aleksandra Danielak, John L Wallace, Tomasz Brzozowski, Marcin Magierowski

**Affiliations:** ^1^ Department of Physiology, Jagiellonian University Medical College, Cracow, Poland; ^2^ Department of Physiology and Pharmacology, University of Calgary, Calgary, AB, Canada

**Keywords:** hydrogen sulfide, carbon monoxide, nitric oxide, non-steroidal anti-inflammatory drugs, gastrointestinal safety, inflammation

## Abstract

Non-steroidal anti-inflammatory drugs (NSAIDs) represent one of the most widely used classes of drugs and play a pivotal role in the therapy of numerous inflammatory diseases. However, the adverse effects of these drugs, especially when applied chronically, frequently affect gastrointestinal (GI) tract, resulting in ulceration and bleeding, which constitutes a significant limitation in clinical practice. On the other hand, it has been recently discovered that gaseous mediators nitric oxide (NO), hydrogen sulfide (H_2_S) and carbon monoxide (CO) contribute to many physiological processes in the GI tract, including the maintenance of GI mucosal barrier integrity. Therefore, based on the possible therapeutic properties of NO, H_2_S and CO, a novel NSAIDs with ability to release one or more of those gaseous messengers have been synthesized. Until now, both preclinical and clinical studies have shown promising effects with respect to the anti-inflammatory potency as well as GI-safety of these novel NSAIDs. This review provides an overview of the gaseous mediators-based NSAIDs along with their mechanisms of action, with special emphasis on possible implications for GI mucosal defense mechanisms.

## Introduction

Non-steroidal anti-inflammatory drugs (NSAIDs), such as aspirin, ketoprofen, naproxen, indomethacin and others, represent one of the most commonly prescribed medications with a wide spectrum of therapeutic effects. Due to their potent analgesic, anti-inflammatory and antipyretic properties they are commonly used to suppress pain, inflammation as well as fever associated with inflammatory diseases including for instance rheumatoid arthritis and osteoarthritis (Bacchi et al., 2012). Additionally, aspirin, being the most widely used antiplatelet drug, remains crucial in the secondary prevention of cardiovascular diseases (Warner et al., 2011), based on its long-term application (Huang et al., 2011). However, despite considerable advantages of NSAIDs, they are known to exert adverse effects including gastrointestinal (GI) - toxicity, cardiovascular complications and renal failure (Varga et al., 2017). In addition, the risk and panel of complications is further increased by comorbidities and longer time of the NSAIDs treatment, which remains a serious limitation in clinical pharmacotherapy (Harirforoosh et al., 2014). Beneficial and adverse effects of NSAIDs are assumed to result from the inhibition of the enzyme cyclooxygenase (COX), leading to the decreased biosynthesis of prostaglandins (PG), hyperalgesic agents (Bacchi et al., 2012). COX is known to exist in two main isoforms – COX-1 and COX-2. COX-1 is constitutively expressed and regulates various physiological functions, whereas COX-2 is considered to be an inducible isoform involved in modulation of pain and inflammatory response under pathological conditions (Conaghan, 2012). However, it should be noted, that this mechanism could be oversimplified in the light of recently published data, showing that COX-2 could be also constitutively expressed in certain tissues of human body (Varga et al., 2017).

GI-adverse effects evoked by NSAIDs are well-documented and involve microbleedings, induction of haemorrhagic lesions, gastroduodenal ulcer formation and even perforations (Lanas, 2010). Two main mechanisms have been implicated in the pathogenesis of these adverse effects, on systemic and local level of NSAIDs activity. The topical action is related to direct toxicity of these compounds toward gastric epithelial cells and mucosal surface, whereas among the systemic effects, the inhibition of PGs biosynthesis leading to an impairment of the organ blood flow is particularly crucial (Wallace, 2008). PGI_2_ and PGE_2_, are mainly produced *via* activity of COX-1 and contribute to the maintenance of gastric mucosal barrier (Konturek, 1985). The following effects have been assumed to be involved in PG-mediated gastroprotection: increased mucosal blood flow, elevated bicarbonate and mucus release, suppression of gastric acid secretion, prevention from leukocyte adherence to the vascular endothelium and downregulation of inflammatory signaling (Wallace, 2008). Therefore, the multifunctional properties of PGs, that embrace both hyperalgesia and GI safety, make this a very challenging prospect to achieve pain and inflammation relief without undesirable GI complications. In recent years many attempts have been undertaken to address this issue and synthesize novel “safer” NSAIDs, which would retain the potent anti-inflammatory action, however, with significantly reduced gastrotoxicity (Sulaieva and Wallace, 2015).

The discovery of second isoform of COX, prompted the development of selective cyclooxygenase-2 inhibitors (coxibs), which were expected to reduce PGs synthesis on the inflammation site, while remaining without such effect in gastric mucosa and thus preserving the potent anti-inflammatory activity with markedly decreased GI-toxicity (Wallace and Muscará, 2001). However, after the enthusiastic introduction of coxibs to the market, serious adverse effects were reported. Although selective COX-2 inhibitors did present improved safety profile in gastric mucosa, generating less ulceration and bleeding, they also exhibited other adverse effects. Precisely, coxibs significantly elevated risk of cardiovascular events such as myocardial infarction, which was a reason for the withdrawal of rofecoxib (Dieppe et al., 2004). This issue raised awareness about important role of COX-2 in the physiological processes in both cardiovascular and renal systems. It has become apparent that inhibition of COX-2 results in suppression of prostacyclin PGI2 synthesis and subsequent imbalance between prothrombotic tromboxane A2 and antithrombotic PGI2, promoting thrombosis and the incidence of cardiovascular events (Mukherjee, 2001). Therefore, despite the preliminary high expectation associated with coxibs and mass promotion of these drugs, they were proved to be of benefit only in a limited group of patients with low risk of cardiovascular death (Topol, 2005).

On the other hand, endogenous gaseous mediators such as nitric oxide (NO), hydrogen sulfide (H_2_S) or carbon monoxide (CO) were shown to be involved in the maintenance of GI integrity, to exhibit gastroprotective action, to accelerate ulcer healing and to modulate gastric blood flow and gastro-duodenal secretion. All three molecules were shown to interact each other within GI tract. H_2_S-, NO- or CO-releasing pharmacological tools and chemicals were reported to prevent gastric mucosa against the damage induced by exposure of GI-mucosa to stress, ischemia/reperfusion, and the mucosal injury caused by pharmacological agents and drugs such as alendronate or aspirin and other NSAIDs. Therefore, over recent years novel H_2_S-, NO- or CO-releasing derivatives of NSAIDs were synthetized (Wallace et al., 2017; Magierowska et al., 2018). Thus, in this review we aimed to provide an update on the recent advances in the development of these novel NSAIDs and their possible molecular mechanisms of action with special emphasis on possible clinical and therapeutic implications.

## Gastroenteropathy Evoked by NSAIDs

**FIGURE 1:**
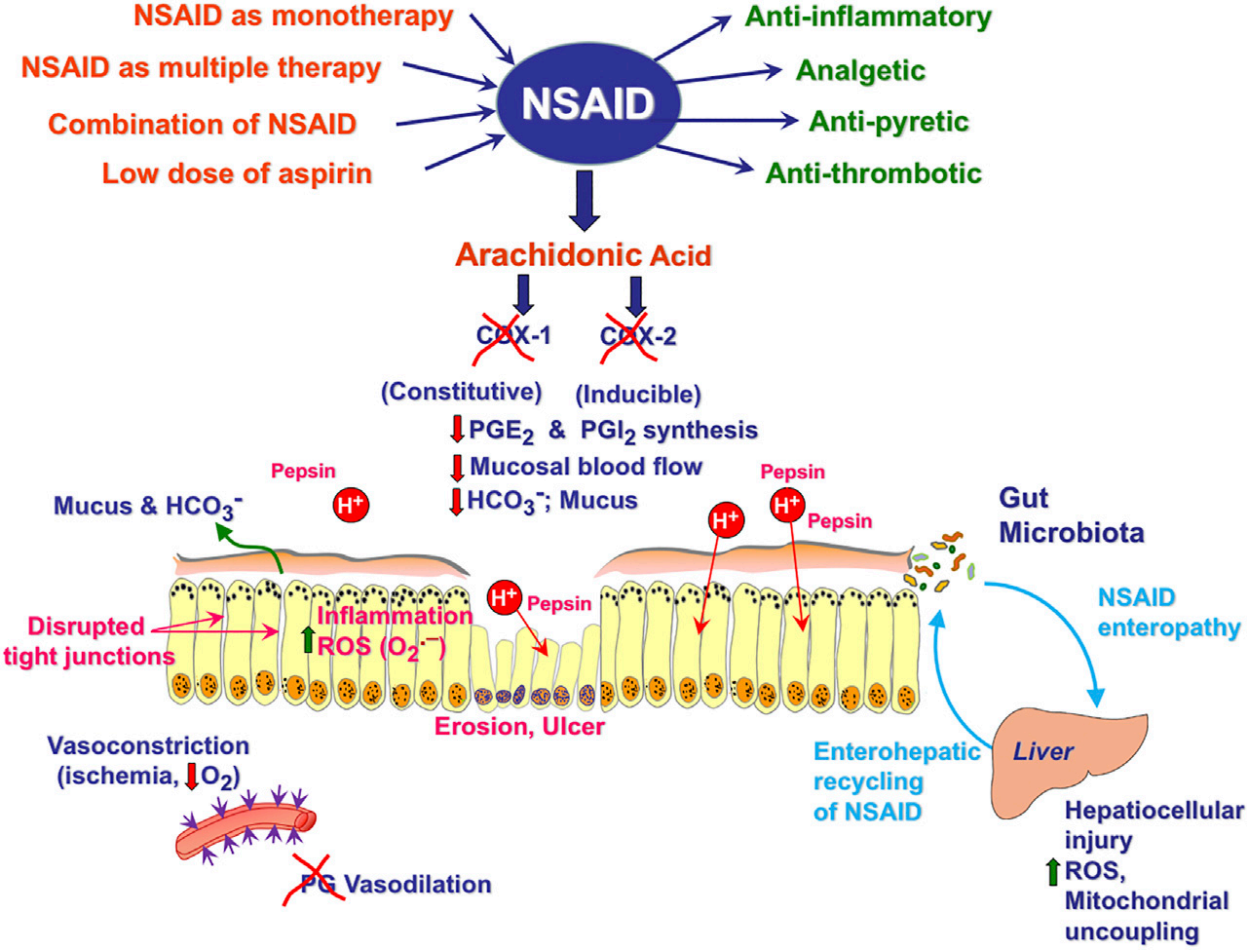
Pathomechanism of NSAIDs-induced gastrointestinal damage.

The gastroduodenal toxicity of NSAIDs has been extensively studied so far and the mechanisms beyond it remain largely described. Theoretically, this already recognized pathomechanism of NSAID-induced damage makes these gastric injuries preventable only to limited extent. Gastric acid has been recognized as a crucial pathogenic factor affecting the weakened gastric mucosa due to NSAID-induced PGs inhibition (Wallace, 2012) (Figure 1). Therefore, the histamine H_2_ receptor antagonists (H2RAs) and proton pump inhibitors (PPIs), both potent acid secretion suppressors have been clinically recommended in prevention of NSAIDs-induced gastric damage (Scheiman et al., 2006). On the other hand, due to the difficulty in diagnosing of small intestine erosions together with its typically subclinical course, NSAID-induced enteropathy constitutes an under-recognized and underestimated clinical problem (Srinivasan and De Cruz, 2017). Development of the small intestine imaging due to the implementation of the capsule endoscopy, revealed that the prevalence of NSAID-induced small intestine injury is far more frequent than it was initially suspected (Graham et al., 2005). Importantly, the incidence of small intestinal damage among healthy subjects receiving NSAIDs combined with PPI over 2 weeks has been estimated at the level of 55–75% (Goldstein et al., 2005; Maiden et al., 2005; Wallace, 2012). As documented by virtual endoscopy, the small bowel injury may be identified as mucosal erythema, erosions, ulcerations and occult GI bleeding, while clinically these damages are manifestated most frequently by iron deficiency anemia (Tai and McAlindon, 2018). Unlike the stomach, there is no proof that gastric acid contributes to the development of intestinal lesions associated with NSAID administration and interestingly, drugs suppressing acid secretion have been shown to even aggravate small bowel injuries (Syer et al., 2015; Tai and McAlindon, 2018). Using capsule endoscopy, Watanabe et al. have reported that among rheumatoid patients treated chronically with NSAIDs, the independent risk factors for severe intestinal damage were the age over 65 years and the use of a PPI along with a H2RA (Watanabe et al., 2013).

Interestingly, the mechanisms underlying the pathogenesis of NSAIDs-enteropathy seem to be complex and distinct from those responsible for gastric damage (Boelsterli et al., 2013). The inhibition of PG biosynthesis has been shown to predispose both, gastric and intestinal mucosa to various injurious factors (Figure 1). However, in contrast to NSAIDs gastropathy, the major mechanism responsible for bowel damage appears to be PG-independent (Matsui et al., 2011). Precisely, the enterohepatic circulation of NSAIDs, bile and enteric microbial flora have been all considered as critical pathogenic factors (Syer et al., 2015) (Figure 1). Enterohepatic recirculation is assumed to depend upon bacterial β-D-glucuronidase, an enzyme that enhances the resorption of NSAIDs in the ileum, leading in consequence to the harmful re-exposure of the intestinal mucosa to these compounds (Wallace, 2013). Importantly, pharmacological inhibition of bacterial β-glucuronidase has been demonstrated in experimental animal model to reduce the re-exposure of these drugs to intestinal epithelium and thus, remarkably alleviate NSAIDs-induced enteropathy (LoGuidice et al., 2012).

It is also of interest that NSAIDs intake has been shown to elicit significant changes in the quality of enteric microbiota, particularly the elevation in the number of Gram-negative bacteria (Wallace, 2012). The pivotal role of microbiota has been additionally emphasized by the observation that germ-free rats were resistant to the indomethacin-induced enteropathy, and this effect was reversed by intestinal contamination with *E.coli* (Robert and Asano, 1977). There is also a compelling body of evidence suggesting that the profound suppression of acid secretion by PPIs affect the number and diversity of intestinal bacteria, contributing to the exacerbation of intestinal damage (Bruno et al., 2019). For instance, when a PPI (omeprazole or lansoprazole) was administered to rats in combination with NSAID, the significant alterations in the enteric microbiome profile were identified, especially manifested by the reduction in the *Bifidobacterium* content, followed by increased severity of intestinal injury (Wallace et al., 2011).

Additionally, bile acid dysmetabolism has been also implicated in the pathogenesis of small bowel damage associated with gastric acid suppression (Blackler et al., 2014). The study by Shindo et al. revealed that the omeprazole treatment in a group of gastric ulcer patients as well as healthy control volunteers resulted in elevated levels of deconjugated bile acids in both groups, which was attributed to the bacterial overgrowth detected in the jejunum (Shindo et al., 1998). Thus, the deconjugation of bile acids due to bacterial enzymatic activity, which is known to enhance the damaging properties of the bile within intestinal epithelium, could also explain the pathogenesis of enteropathy mediated by long-term acid inhibition due to PPI treatment.

To summarize, until now there is no successful strategy being sufficiently proven to offer a fully effective therapeutic approach either in the prevention or in the treatment of NSAIDs gastroenteropathy. Therefore, there is definitely a need for the development of novel effective therapeutic approaches in this field.

## Gaseous Mediators-Releasing NSAIDs and GI Tract

### NO-Releasing NSAIDs

NO is a gaseous mediator involved in the regulation of numerous physiological pathways including those responsible for the homeostasis of the GI tract. NO is endogenously produced within esophageal, gastric and intestinal mucosa *via* the enzymatic activity of NO synthases:

• neuronal (nNOS), expressed in the neurons of central and peripheral nervous system,
• endothelial (eNOS) located in both endothelial cells and platelets,
• inducible (iNOS), which is expressed in endothelial cells, smooth vascular muscle, neutrophils and macrophages.

**FIGURE 2:**
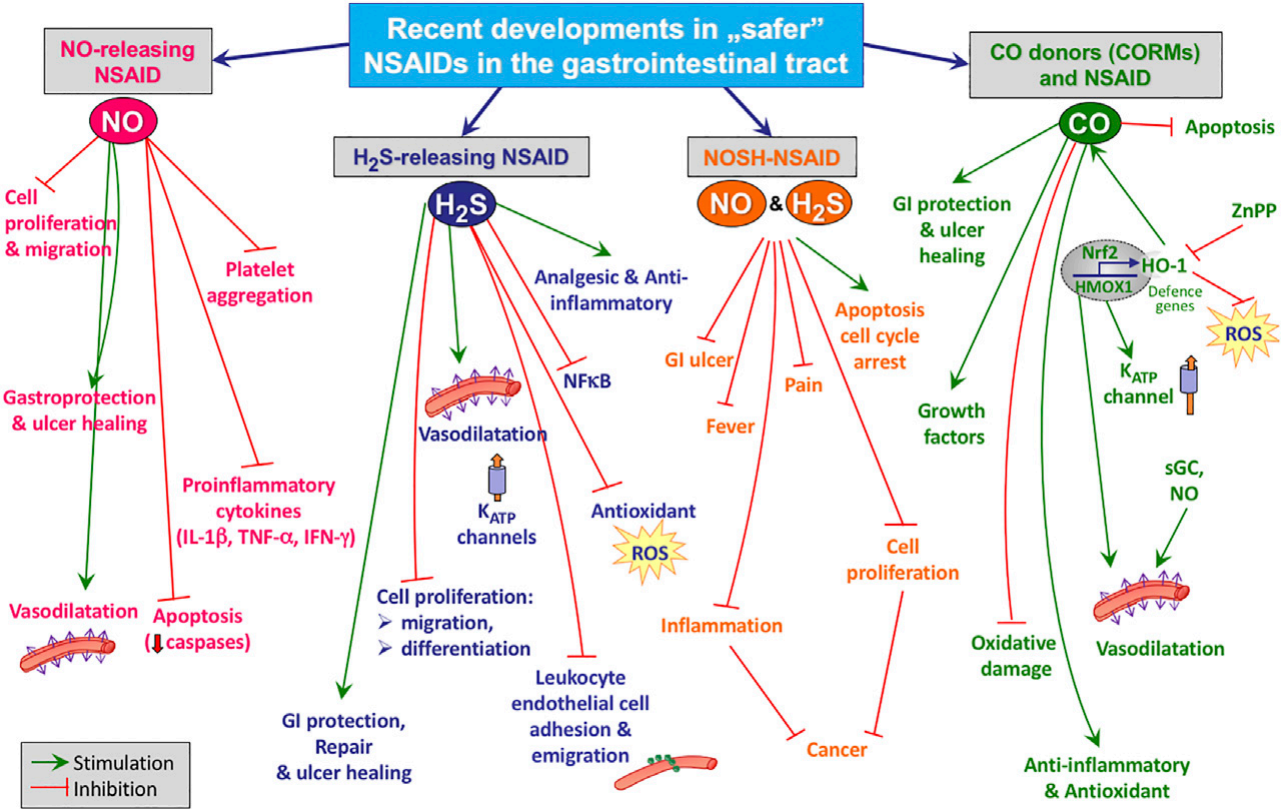
Possible mechanisms of action of NO-, H2S-releasing NSAIDs, NOSH-NSAIDs or NSAIDS applied with CO donors.

Both nNOS and eNOS are constitutive isoforms responsible for the maintenance of GI mucosal defense due to the constant generation of small amounts of NO (Stanek et al., 2008). According to the recent data, the beneficial actions of NO in GI tract include vasodilatation, crucial for the maintenance of blood flow and mucosal barrier integrity, the enhanced epithelial mucus and bicarbonate secretion, the reduced adherence of neutrophils and their infiltration of gastric tissue resulting in diminished reactive oxygen species (ROS) production and decreased oxidative stress (Figure 2) (Magierowski et al., 2015a). Furthermore, NO has been proven to regulate the gastric and intestinal epithelial cells tight junctions through mechanisms involving inhibition of protein and lipid oxidation, modulation of glutathione (GSH/GSSG) ratio, which is crucial for cell survival, and the maintenance of redox homeostasis (Mu et al., 2019). On the other hand, a decrease in NO production has been implicated in the pathogenesis of many diseases including *diabetes mellitus*, hypertension and arteriosclerosis. Moreover, it has been shown that the pharmacologic inhibition of NO-synthase impaired the healing rate of gastric ulcers (Magierowski et al., 2015).

Based on these physiological and pharmacological properties of NO, a new class of NO-releasing NSAIDs (NO-NSAIDs) was developed by chemically binding a NO-releasing moiety with the parent drug (Fiorucci et al., 2001). Importantly, NO-NSAIDs have been shown to exert anti-inflammatory and analgetic effects comparable or even greater to those provided by conventional NSAIDs with markedly lower GI toxicity (Wallace et al., 1995; Wallace et al., 1999; Al-Swayeh et al., 2000; Kato et al., 2001; Fiorucci et al., 2003; Fiorucci et al., 2004; Rolando et al., 2013). The anti-inflammatory activity of NO-NSAIDs have been proved by adding the NO moiety to various derivatives including aspirin, naproxen, ketoprofen, flurbiprofen, ibuprofen, diclofenac, indomethacin, mesalamine, tolfenamic acid, or celecoxib (Pereira-Leite et al., 2017). It is worth mentioning that NCX-4016 (NO-aspirin) and NCX-530 (NO-indomethacin) have been shown to protect gastric mucosa against HCl/ethanol-induced damage (Takeuchi et al., 1998; Takeuchi et al., 2001). Interestingly, in a rodent model, the administration of NCX-530 vs parent indomethacin did not evoke intestinal damage, and the inhibition of bacterial translocation within intestine with this novel NO-NSAID agent has been proposed to explain this phenomenon (Mizoguchi et al., 2001). Additionally, it has been demonstrated, that the prolonged administration of NO-NSAIDs in contrast to classic parent NSAIDs do not delay, but even accelerate the healing of gastric ulcers (Elliott et al., 1995; Brzozowski et al., 2000; Brzozowska et al., 2004). It should be also noticed, that NCX-4016 and NCX-4215, being another derivative of aspirin releasing NO, have been shown to exert increased antithrombotic activity, comparable to that exhibited by aspirin (Wallace et al., 1995; Momi et al., 2000; Bolla et al., 2006).

These promising results obtained in pre-clinical animal studies provided significant evidence to further explore the assessment of NCX-4016 and nitronaproxen (naproxcinod) in humans. NCX-4016 completed phase 1 clinical trial and entered phase 2, however the further development of this novel NO-NSAID was terminated due to the detected genotoxicity (Di Napoli and Papa, 2003). Naproxcinod successfully completed phase 3 clinical trial and has been shown to be effective in relieving the symptoms of osteoarthritis, while having significantly reduced GI toxicity (Lohmander, 2004; Schnitzer et al., 2005; KARLSSON et al., 2009).

**FIGURE 3:**
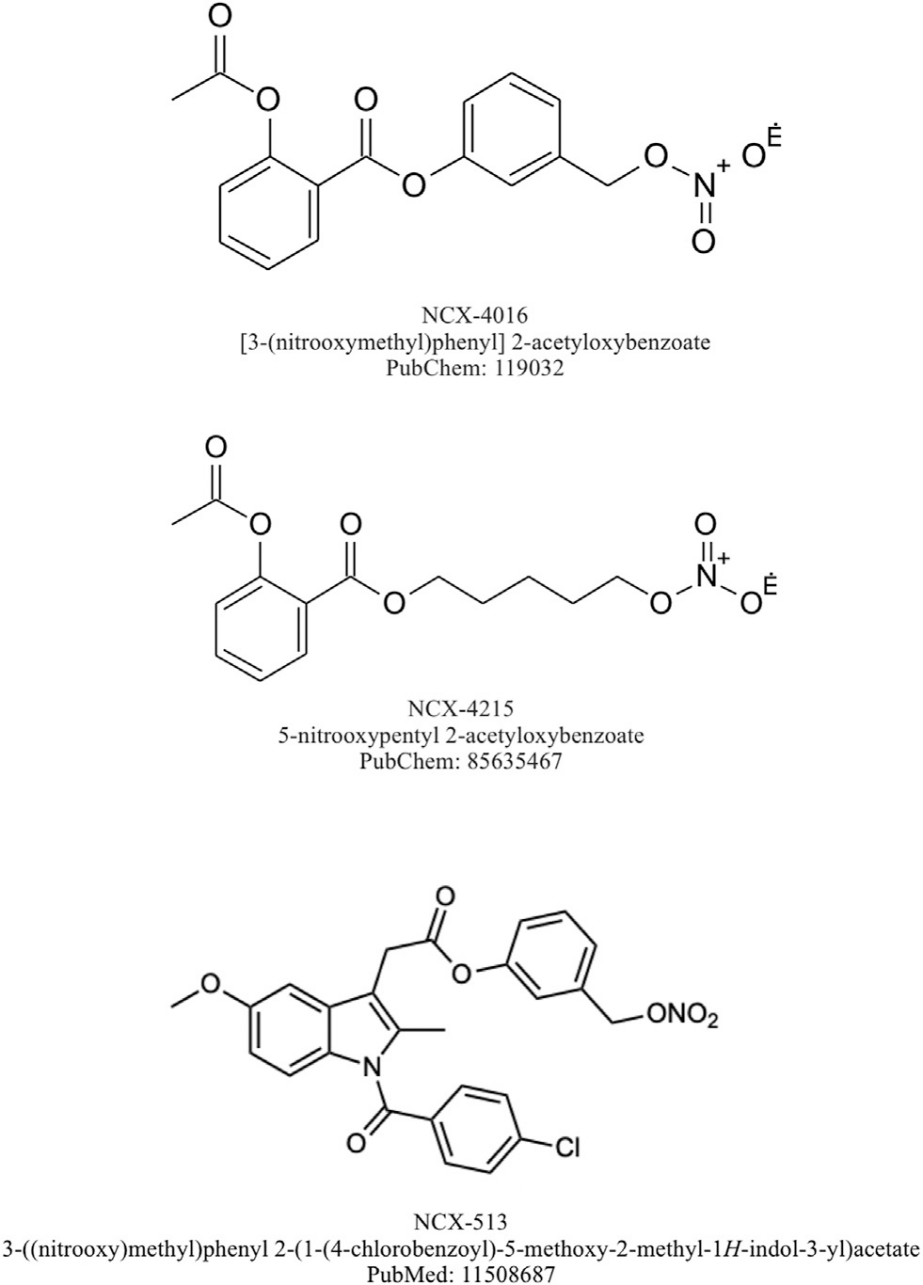
Chemical structures of selected NO-NSAIDs.

Taking into account that NSAIDs-based pharmacotherapy among elderly diabetic patients correlates with the higher risk of adverse effects such as impaired renal function, excessive fluid retention and aggravated hypertension (Caughey et al., 2010), NO-NSAIDs seemed to be promising alternative but with one essential adverse effect such as the increased risk of NO-mediated hypotension. Pieper et al. (Pieper et al., 2002) documented in their animal study, the protective effect of chronic NCX-4016 treatment against impaired endothelium-dependent relaxation, which is an important feature of human *diabetes mellitus*. Similarly, in another report NCX-4016 treatment in the group of diabetic rats resulted in significantly reduced vascular endothelium damage (Ambrosini et al., 2005). Clearly, the further studies on the usage of NO-NSAID among humans with diabetes are needed to shed more light into the potentially beneficial effects of these novel drugs to counteract some vascular and epithelial aspects of metabolic disorders. Chemical structure of selected NO-NSAIDs are presented on Figure 3.

### H_2_S–Releasing NSAIDs

H_2_S, similarly to NO, is an important endogenous gaseous messenger known to modulate the cardiovascular and nervous system functioning, GI defense as well as the inflammatory pathways (Bełtowski, 2015). It is produced within human body due to activity of two enzymes: cystathionine- γ-lyase (CTH) and cystathionine-β-synthetase (CBS) (Huang and Moore, 2015). H_2_S could be also generated in mitochondria by the activity mercaptopyruvate sulfurtransferase (MPST) (Kimura, 2014). Additionally, this molecule could be produced and metabolized by microbiota within the gut. The endogenous and exogenous H_2_S has been recognized as an anti-inflammatory, anti-oxidative and vasodilatory agent, essentially involved in the modulation of inflammatory cascade and the process of resolution of inflammation (Figure 2.) (Wallace, 2010). Furthermore, endogenous and exogenous H_2_S released from chemical donors have been implicated in the mechanism of maintenance of gastric mucosal homeostasis and to protect the gastric mucosa against lesions induced by NSAIDs and other noxious factors (Lou et al., 2008; Cipriani et al., 2013; Magierowski et al., 2015; Magierowski et al., 2016; de Araújo et al., 2018; Magierowski et al., 2018). Such a protective effect of H_2_S has also been demonstrated in the intestinal mucosa of NSAID-treated rats, which seems to be particularly important due to the current need for an effective therapy against the adverse effects of NSAIDs affecting the intestine (Wallace and Wang, 2015).

A remarkable progress has recently been achieved in the understanding of mechanisms underlying the protective action of H_2_S within GI tract, by demonstration that this gaseous molecule enhanced mucosal microcirculation, attenuated the TNF-α signaling along with suppression of pro-inflammatory cytokines expression and leukocyte adherence (Wallace, 2010). Importantly, H_2_S released form chemical donors such as NaHS or Lawesson’s reagent, as emphasized by many experimental animal studies, decreased the number and severity of gastric mucosal lesions evoked by NSAIDs, ischemia/reperfusion, ethanol and stress (Zanardo et al., 2006; Mard et al., 2012; Magierowski et al., 2015; Magierowski et al., 2016; Sun et al., 2017; Magierowski et al., 2018).

On the other hand, the inhibition of endogenous H_2_S production was associated with remarkably elevated adherence of leukocytes to the vessels wall and with aggravated inflammation accompanying induction of paw edema in rats (Zanardo et al., 2006). The study by Fiorucci et al. (2005) revealed that deleterious gastric adverse effects evoked by NSAIDs may be to some extent ascribed to the suppression of endogenous H_2_S biosynthesis, which was documented by a decreased CTH mRNA and protein expression during NSAID treatment.

Therefore, numerous derivatives of H_2_S-releasing NSAIDs have been developed with improved safety profile of these compounds compared with parent drugs documented in preclinical studies (Magierowski et al., 2015). Promising effects have been achieved with respect to the following novel NSAIDs: H_2_S-releasing naproxen (ATB-346), H_2_S-releasing diclofenac (ATB-337 and ACS-15), H_2_S-releasing ketoprofen (ATB-352) and H_2_S-releasing aspirin (ATB-340 and ACS-14). Administration of ATB-337 evoked approximately 90% less intestinal damage, when compared to native diclofenac (Wallace et al., 2007). The possible mechanisms responsible for preventive activity of hydrogen sulfide within intestine seem to involve, at least partially, improvement of intestinal barrier, facilitation of antimicrobial immunity and normalization of enteric microbiota profile (Schroeder et al., 2011; Blackler et al., 2015).

Wallace et al. (Wallace et al., 2007) reported that ATB-337 was less gastrotoxic and produced remarkably less injury within gastric as well as intestinal mucosa in rats as compared to its parent drug. Furthermore, ATB-337 was more effective in decreasing of paw edema evoked by carrageenan. Similarly, S-diclofenac (ACS-15) exhibited augmented anti-inflammatory activity against LPS-induced inflammation in comparison with the parent drug, which could be due to: 1) enhanced ability of S-diclofenac to inhibit LPS-induced upregulation of iNOS expression, 2) Nf-κB pathway suppression – the effect which was not observed in diclofenac treatment (Li et al., 2007). Liu et al. (2012) have demonstrated that H_2_S-releasing aspirin (ACS-14) decreased the gastric lesions formation induced by aspirin administration and restored H_2_S plasma level, which was substantially reduced in rats subjected to acetylsalicylic acid. Also, H_2_S-releasing ketoprofen (ATB-352) reduced the inflammation and concomitant bone resorption in rats in experimental model of periodontitis. This effect was accompanied by a significantly diminished incidence of gastric lesions as compared to rats pretreated with classic ketoprofen (Gugliandolo et al., 2018). In addition, Glowacka et al. (2021) reported that GI toxicity was significantly reduced during chronic treatment with ATB-352 in rats, when compared to parent drug. Furthermore, ketoprofen affected intestinal microbiome profile to much greater extent than ATB-352 and, in sharp contrast to ATB-352, required co-therapy with omeprazole to counteract detrimental alterations within GI tract. Therefore, novel H_2_S-releasing ketoprofen could provide improved therapeutic strategy, preventing from the necessity of co-treatment of NSAIDs with PPIs and, in consequence, further impairment of intestinal microbiome induced by PPIs.

**FIGURE 4:**
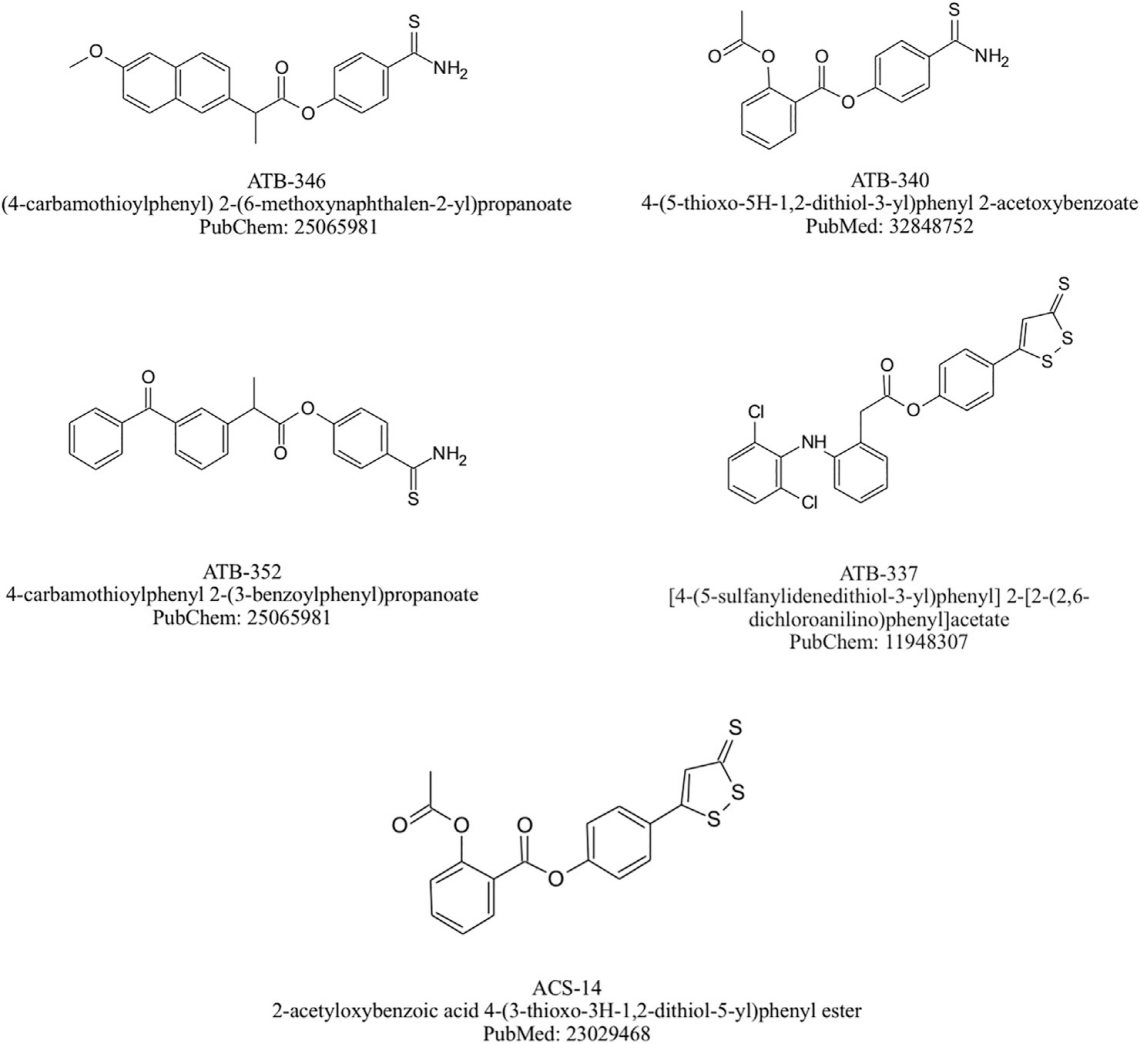
Chemical structures of selected H_2_S-NSAIDs.

Nevertheless, ATB-346 could be considered as the leading drug in this class. This H_2_S-releasing derivative of naproxen has been designed for osteoarthritis therapy and it has been confirmed by many animal studies to be as effective as the equimolar doses of its parent drug in terms of COX-1 activity inhibition leading to decreased PGE_2_ biosynthesis and alleviation of inflammatory response with significantly decreased GI-toxicity (Blackler et al., 2012; Ekundi-Valentim et al., 2013; Sulaieva and Wallace, 2015; Wallace and Wang, 2015; Magierowski et al., 2017; Van Dingenen et al., 2019). Interestingly, ATB-346 was shown to exert therapeutic effect reflected by the acceleration of gastric ulcers healing in contrast with its parent drug, known not only to exacerbate acute gastric mucosal lesions but also producing a delay in the ulcer healing (Magierowski et al., 2015). Noteworthy, the mechanism of therapeutic properties of this compound has been assumed to involve activation of Nrf-2/HMOX-1/CO pathway, responsible for increased endogenous CO production, since it was observed that protein expression of Nrf-2 and HMOX-1 was significantly elevated in gastric mucosa of rats administered with ATB-346 (Magierowski et al., 2017). Blackler et al. (2012) pointed out that adverse effects of NSAIDs occur especially frequently among elderly patients with co-existing health problems, while most of the studies evaluating the safety of novel drugs is conducted on healthy animals. Based on this, they compared the possible outcomes of ATB-346 and NCX-429 (NO releasing derivative of naproxen) administration among rats afflicted with arthritis, obesity, or hypertension. This study revealed that both novel compounds not only presented similar anti-inflammatory properties comparing to their parent drugs – naproxen and celecoxib, but also they did not evoke mucosal damage within GI tract, suggesting their safety and tolerability by individuals with co-morbidities (Blackler et al., 2012). Recent animal studies have also reported that ATB-346 exerts protective effect on neuroinflammation, neural cell death and brain oxidative stress and may have therapeutic potential to treat traumatic brain injury or neurodegenerative diseases such as Alzheimer’s disease (Campolo et al., 2014; Mostafa et al., 2016). Importantly, a phase I clinical trial revealed the safety and good tolerability of ATB-346 (administered in doses ranging from 25 to 2,000 mg). This observation was associated with the significantly lower risk of adverse effects, comparable to that observed in placebo group (Wallace et al., 2018). Interestingly, the plasma half-life of naproxen turned out to be substantially longer in group of patients receiving ATB-346. Recently, ATB-346 has also completed Phase II clinical trial and presented significantly lower rate of GI-tract adverse effects such as ulceration, gastroesophageal reflux, abdominal discomfort or dyspepsia when compared to naproxen (Wallace et al., 2020). Chemical structure of selected H_2_S-NSAIDs are presented on Figure 4.

### NOSH-NSAIDs

Keeping in view the therapeutic properties of NO and H_2_S, new compounds integrating both these gaseous mediators have been developed, namely NOSH-NSAIDs. Recent reports documented the promising effects of NOSH-NSAIDs with respect to the growth inhibitory properties in various human cancer cell lines, such as colon, pancreatic, breast, lung, prostate and leukemia cancer cells (Figure 2) (Chattopadhyay et al., 2012; Kodela et al., 2012; Kodela et al., 2013; Kashfi, 2015; Kashfi et al., 2015; Vannini et al., 2015a; Chattopadhyay et al., 2020). Interestingly, several studies demonstrated enhanced chemopreventive potential of these novel hybrids compared with parent drugs. For instance, NOSH-ASA was more effective than ASA in HT-29 colon cancer cells (Kodela et al., 2012), whereas NOSH-naproxen (AVT219) was shown to possess improved ability in suppressing the growth of adenomatous, epithelial, and lymphocytic cancer cell lines, as compared to naproxen (Chattopadhyay et al., 2016). Also, a study by Kodela et al. (2013) identified NOSH-naproxen as approximately 8000-fold more effective than the combination of its components in prevention from the growth of human colon cancer cells, implying a remarkable synergistic effect. A similar trend was observed with NOSH-ASA (NBS-1120), being 9000-fold more potent than the sum of its partial molecules in suppressing cell growth (Chattopadhyay et al., 2012). Furthermore, efficacy of NOSH-NSAIDs to influence cancer growth, seems to be dependent on the induction of cell cycle arrest and apoptosis, along with increase in reactive oxygen species (ROS) level, known to promote cell death (Vannini et al., 2015b). Importantly, several animal studies have shown that NOSH-ASA, NOSH-sulindac (AVT-18 A) and NOSH-naproxen, in contrast to their parent drugs, were devoid of GI complications such as ulceration and bleeding, which clearly indicates improved safety profile of these novel compounds (Kashfi, 2015; Chattopadhyay et al., 2016; Chattopadhyay et al., 2020). Additionaly, Kashfi et al. (2015) have demonstrated using a rat model that administration of NOSH-sulindac was associated with decreased lipid peroxidation and increased SOD activity, being an antioxidant marker, in gastric mucosa, as compared to the parent drug sulindac.

On the other hand, NOSH-NSAIDs have also attracted attention as anti-inflammatory agents and as documented in preclinical studies using the carrageenan rat paw edema model, these novel NO and H_2_S releasing derivatives exerted similar or even enhanced anti-inflammatory potential than their parent compounds (Kodela et al., 2013; Fonseca et al., 2015; Kashfi, 2015; Kashfi et al., 2015). Furthermore, anti-pyretic, analgesic and anti-platelet properties of NOSH-NSAIDs were comparable to those evoked by conventional NSAIDs (Kashfi et al., 2015). However, we assume that there is a lack of the studies directly comparing these NOSH-NSAIDs not only to the parent NSAID, but also to the respective NO-NSAID and H_2_S-NSAID to evaluate whether combining the two gaseous molecules really brings further improvement over the single mediator-releasing NSAID.

**FIGURE 5:**
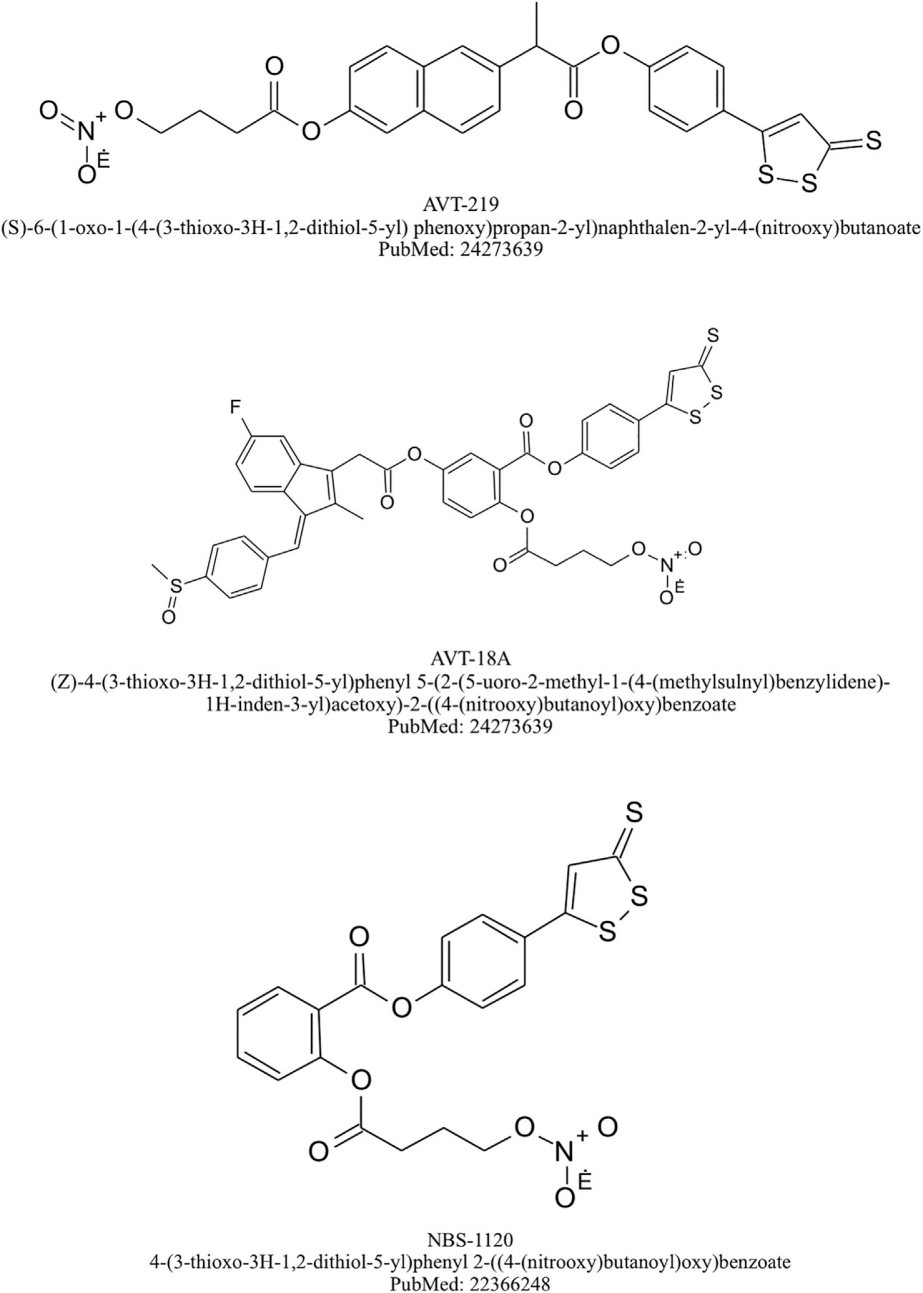
Chemical structures of selected NOSH-NSAIDs.

Noteworthy, NOSH-NSAIDs have shown promising results in attenuating neuroinflammation and preventing from neuronal death in animal model of ischemic stroke (Lee et al., 2013; Ji et al., 2017). Interestingly, in a human cell model of neuroinflammation the neuroprotective activity of NOSH-ASA was remarkably more potent than NO- or H_2_S-releasing ASA, which is consistent with previous observation on synergistic effect of NOSH-NSAIDs (Lee et al., 2013). Thus, NOSH-NSAID may provide new strategies for the treatment of neurodegenerative disorders comprising Alzheimer’s disease and Parkinson’s disease. Chemical structure of selected NOSH-NSAIDs are presented on Figure 5.

### CO-Releasing Molecules

CO is perceived to be a poisonous 'silent killer' gaseous molecule but it is now receiving increasing attention as an important endogenous messenger constantly produced in small amounts within human body (Wu and Wang, 2005). CO is generated by degradation of free heme to biliverdin *via* the enzymatic activity of heme oxygenases (HMOX) - constitutive HMOX-2 highly expressed in brain and testes, and inducible isoform HMOX-1 (which is also known as heat shock protein 32 – HSP 32), reported to be activated in response to various stressful stimuli such as LPS, inflammatory cytokines or oxidative stress and plays a crucial role in the maintenance of cellular homeostasis (Choi and Alam, 1996; Higuchi et al., 2009; Takahashi et al., 2009). The well-known cellular targets of CO are soluble guanylyl cyclase (sGC) producing cGMP, cytochrome p-450 and other cytochromes, nitric oxide synthase (NOS), and possibly COXs (Magierowski et al., 2017; Ryter et al., 2018). Importantly, CO seems to upregulate the production of NO in remote tissues suggesting that its supplementation may represent better strategy than molecules liberating NO, which is incapable of being transported to distant targets because of its high reactivity (Motterlini and Otterbein, 2010).

Currently, more light is being shed regarding regulatory functions of CO in many systems including cardiovascular, GI and nervous systems and therapeutic effect of CO-donors on various diseases such as atherosclerosis, hypertension, diabetes, chronic pulmonary disease or gastric ulcers (Motterlini and Otterbein, 2010; Jasnos et al., 2014; Olas, 2014). There is growing data emphasizing potent anti-inflammatory and cytoprotective properties of CO, known to be dependent on multiple pathways involving mitogen-activated protein kinase (MAPK), the elevated cGMP level, the enhanced expression of antioxidant enzymes, and the downregulation of inflammatory Nf-κB pathway (Figure 2) (Qin et al., 2015; Sulaieva and Wallace, 2015; Ling et al., 2018).

In the light of these discoveries, several CO-prodrugs, namely CO-releasing molecules (CORMs) were developed to provide safe, manageable way of delivering physiologically efficient quantities of exogenous CO to various tissues and organs. The range of CORMs reported so far is wide and comprises the most investigated ones [Mn2(CO)10] (CORM-1), tricarbonyldichlororuthenium (II) dimer [Ru(CO)3Cl2]2 (CORM-2) and tricarbonylchloro (glycinato)ruthenium (II) (CORM-3) (Kautz et al., 2016). Numerous animal studies have confirmed that CO released from CORMs affords gastroprotection against gastric mucosal injury induced by ethanol, ischemia/reperfusion, stress or NSAIDs and has a beneficial influence by acceleration of gastric ulcers healing (Magierowska et al., 2016; Takagi et al., 2016; Magierowski et al., 2017; Magierowska et al., 2018; Magierowska et al., 2019; Magierowska et al., 2019). What is more, Freitas et al. (2006) have documented in their mice model of acute mesenteric inflammation evoked by carrageenan administration, that dimagnese decacarbonyl DMDC, a CO donor, remarkably decreased leukocyte adhesion and migration to the inflamed tissue and this effect was reversed by a soluble guanylate cyclase (sGC) inhibitor.

**FIGURE 6:**
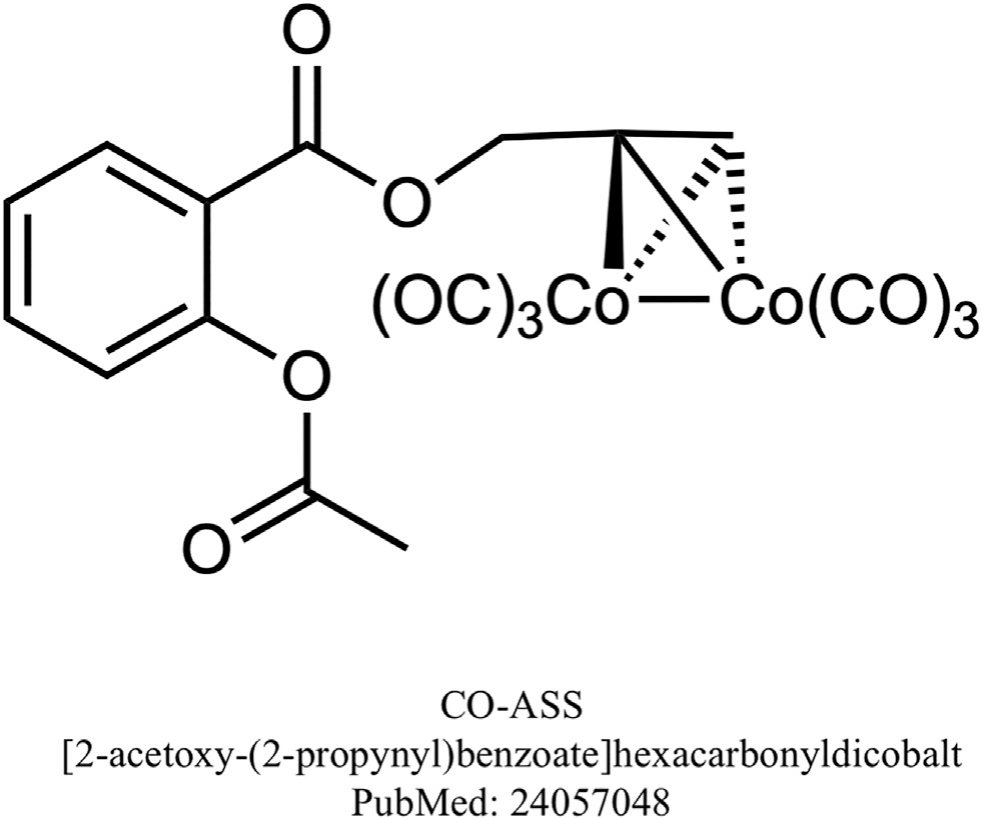
Chemical structure of CO-ASS.

Furthermore, combined administration of CORM-2 with aspirin decreased gastric lesion index, increased gastric blood flow (GBF) and attenuated gastric mucosal lipid peroxidation when compared to aspirin administered alone (Magierowski et al., 2018). In another study pretreatment with CORM-2 alleviated aspirin-induced gastric mucosal damage and this beneficial effect was partly dependent on endogenous NO synthesis, but not on H_2_S pathway (Magierowski et al., 2016; Magierowska et al., 2018). Interestingly, also novel metal-free organic CO-prodrug, BW-CO-111 has been reported to prevent aspirin-induced gastric damage (Bakalarz et al., 2021). Despite promising effects of combined treatment with CORMs and NSAIDs in order to limit NSAID-induced gastrotoxicity, the chemical synthesis of CO-releasing NSAIDs has not been successful and there is little data published and available on this class of prodrugs. However, the effect of CO-releasing aspirin derivative - (CO-ASS) on malignant pleural mesothelioma (MPM) cell lines was investigated and CO-ASS exerted antiproliferative effect and inhibition of Nf-κB (Zanellato et al., 2013). It was hypothesized by the authors that CO-ASS might be of benefit when used as a CO releasing NSAID novel therapeutic, nevertheless further studies are needed to confirm this theory. Chemical structure of CO-ASS is presented on Figure 6.

## Cardiovascular and Renal Safety of Gaseous Mediators-Releasing NSAIDS

Despite the initial focus on GI adverse effects of NSAIDs, their deleterious impact on cardiac, vascular and renal systems has gained increasing attention, especially when coxibs were introduced and their serious cardiovascular adverse effects were detected. Subsequent studies have confirmed that the widespread use of NSAIDs may be associated with harmful cardiovascular and renal complications including hypertension, myocardial infarction, heart failure as well as acute kidney injury and chronic kidney disease (Bindu et al., 2020). Importantly, NSAIDs have been shown to counteract the effects of antihypertensive drugs, in mechanisms involving PGs biosynthesis inhibition, leading to increased arterial blood pressure (Fournier et al., 2012). Also, given that the usage of NSAIDs is known to advance among elderly patients due to multiple diseases, when at the same time renal function tends to deteriorate in age-related mechanisms, this group seems to be especially vulnerable to renal toxicity (Cabassi et al., 2020). Cardiovascular and renal adverse effects of NSAIDs constitute therefore a serious limitation to clinical application of this class of drugs, particularly among patients with numerous comorbidities within GI tract, kidneys and cardiovascular system. On the other hand, promising results have been observed with respect to the cardiovascular and renal safety profile of gaseous mediators-releasing NSAIDs. Interestingly, these novel compounds have been even shown to afford protection against various diseases affecting cardiac, vascular and renal systems.

It has been hypothesized that NO-NSAIDs, due to the ability to release NO, could exert therapeutic effects in diseases associated with reduced NO bioavailability such as diabetes mellitus, known to induce endothelial dysfunction (Shi and Vanhoutte, 2017). Consistent with this assumption, it has been demonstrated by Pieper et al. (2002) that NO-donating aspirin NCX-4016 reduced the development of impaired endothelium-dependent relaxation in animal model of chronic diabetes mellitus. Importantly, the endothelium dysfunction has been recognized as a marker of elevated risk of atherosclerosis and cardiovascular events (Bonetti et al., 2003). Additionally, NCX-4016 administration was accompanied by a decrease in plasma isoprostanes levels, suggesting that therapeutic effect of this compound may be related to its antioxidant activity, possibly by NO-dependent reduction of lipid peroxidation. Interestingly, prolonged treatment with NCX-4016 did not induce a nitrate tolerance, which constitutes a pivotal limitation to clinical use of NO-donors such as nitrates (Pieper et al., 2002). Moreover, experiments carried out on isolated rat aortas showed that NO-NSAIDs such as NCX-4215, nitroflurbiprofen and nitroparacetamol caused dose-dependent relaxation of aortic rings precontracted with adrenaline or noradrenaline (Del Soldato et al., 1999; Keeble et al., 2001). Interestingly, this effect seemed to be endothelium-independent, because removal of endothelium even potentiated vasodilatatory properties of nitroflurbiprofen, possibly due to enhanced impact on the smooth muscle cells layer (Keeble et al., 2001). Furthermore, recent animal studies have indicated that NO-NSAIDs are devoid of hypotensive side effects, known to be a common complication during nitrates therapy (Wallace et al., 1995; Fujihara et al., 1998; Keeble et al., 2001). Possible explanation could be that NO-NSAIDs liberate NO at a slower rate in comparison to standard NO donors (Wallace et al., 1995). On the other hand, interesting results have been obtained by Muscará et al. (2001) who reported that NCX-4016 significantly reduced increased arterial blood pressure in hypertnesive rats, but did not affect the blood pressure in normotensive animals. Thus, considering the fact that administration of standard NSAIDs is often complicated by impairment in the efficacy of antihypertensive drugs, NCX-4016 could be promising alternative for patients with hypertension, who require prolonged NSAIDs therapy (Muscará et al., 2001). Moreover, it has been found that pretreatment with NCX-4016 resulted in significant cardioprotection *in vivo* in a model of acute myocardial ischemia as well as *in vitro* based on the model of ischemia/reperfusion myocardial injury, while control treatment with aspirin showed only little or no protection (Rossoni et al., 2000; Rossoni et al., 2001). In detail, NCX-4016 was shown to markedly reduce infarction size and to decrease plasma activity of creatine kinase, which was accompanied by a significant fall in mortality rate (Rossoni et al., 2000; Rossoni et al., 2001). In addition, chronic treatment with NCX-4016 has been demonstrated to exert anti-atherosclerotic and anti-oxidative activity in arteries of hypercholesterolemic mice (Napoli et al., 2002). On the other hand, Napoli et al. (Napoli et al., 2001) investigated the effect of NCX-4016 on the extent of restenosis after balloon angioplasty in mice with concomitant hypercholesterolemia. They revealed that NCX-4016 showed both preventive and therapeutic potency against restenosis, suggesting that it may be a novel therapeutic strategy in reducing restenosis among patients subjected to balloon angioplasty, especially those with hypercholesterolemia (Napoli et al., 2001). Importantly, NCX-4016 has also shown protective effects in a human trial conducted among patients with intermittent claudication, pathology in which physical effort induces endothelial dysfunction in ischemia/reperfusion-dependent mechanisms. NCX-4016 administered orally for four weeks, significantly prevented from the exercise-induced endothelial damage, in contrast to the standard aspirin treatment, which was devoid of such effect (Gresele et al., 2007). On the other hand, another human trial revealed that NCX-4016 markedly reversed insulin resistance and enhanced vascular response to insulin in obese insulin-resistant men (Gresele and Momi, 2006). Clearly, further studies are required to fully evaluate the clinical potential of novel NO-NSAIDs with respect to cardiovascular disorders.

It has been recently demonstrated that also CO and H_2_S released from chemical donors may provide protection within the cardiovascular system. Numerous studies have shown that CO- and H_2_S-releasing prodrugs exert vasodilatatory and antihypertensive properties (Motterlini et al., 2002; Foresti et al., 2004; Li et al., 2008; Rossoni et al., 2010; Failli et al., 2012; Kulkarni-Chitnis et al., 2015). Rossoni et al. (2010) evaluated the efficacy of H_2_S-releasing derivatives of aspirin (ACS-14) and salicylic acid (ACS-21) in modulation of pathological changes related to metabolic syndrome induced by glutathione (GSH) depletion in rats. ACS-14 and ACS-2 but not classic aspirin, significantly restored endothelial dysfunction in aortic tissue and markedly reduced accompanying hypertension along with hyperinsulinemia, most probably in mechanisms dependent on H_2_S release (Rossoni et al., 2010). On the other hand, experiments carried out on healthy dogs revealed that administration with H_2_S-releasing naproxen, ATB-346, did not produce significant change in arterial blood pressure or heart rate (Wallace et al., 2018). Similarly, phase 1 clinical trial of ATB-346 demonstrated no significant difference in blood pressure between group treated with ATB-346 and placebo control (Wallace et al., 2018). Thus, further research should shed more light into understanding the mechanisms by which H_2_S could affect blood pressure under physiological and pathological conditions.

Interestingly, CO-releasing molecules, CORMs, have been also pointed out as potential antihypertensive agents. Preclinical studies demonstrated that treatment with CORM-3 induced relaxation of precontracted aortic rings *ex vivo*, leading to significant vasodilatation with the involvement of potassium channels and guanylate cyclase activity (Motterlini et al., 2002; Foresti et al., 2004; Failli et al., 2012). Additionally, in response to CORM-3 administration, a significant decrease in blood pressure *in vivo* in rats has been noticed, suggesting possible use of CO-donating compounds as alternative therapy for hypertension (Foresti et al., 2004). This seems to be supported by the observation that CORMs showed efficacy to attenuate acute hypertension in animal models (Motterlini et al., 2002). Also, numerous studies have presented protective effects of H_2_S-NSAIDs, GYY4137 as chemical donor slowly liberating H_2_S, and CORMs against myocardial injury induced by ischemia/reperfusion (Clark et al., 2003; Guo et al., 2004; Varadi et al., 2007; Rossoni et al., 2008; Citi et al., 2018). It is also worth mentioning that in another animal study GYY4137 attenuated atherosclerotic lesions in apoE (-/-) mice, which was accompanied by a significant decrease in vascular inflammation and oxidative profile (Liu et al., 2013). This data seems to be consistent with the observation that atherosclerosis correlates with decreased vascular H_2_S level (Liu et al., 2013). Thus, it could be of interest to determine whether H_2_S-releasing NSAIDs could be considered as therapeutic agents against atherosclerotic vascular lesions. On the other hand, the clinical implementation of CORMs may by limited due to the risk of cardiomyopathy (Kim and Choi, 2018). Therefore, further studies are still required to fully elucidate the influence of CO released from chemical donors on cardiac tissue.

As it has been mentioned above, chronic treatment with NSAIDs also increases the risk of various forms of kidney injury and concomitant reduction in renal perfusion, secondary to decreased PGs biosynthesis (Whelton, 1999). However, gaseous mediators-releasing NSAIDs could be devoid of renal adverse effects and even exert protective activity against kidney disorders. Wallace et al. (1997) reported that administration of diclofenac to healthy or cirrhotic rats resulted in significant reduction in renal blood flow, while NO-releasing derivative of this drug did not induce such effect. Also, phase 1 clinical trial did not show any significant renal side effects among healthy subjects during therapy with H_2_S-releasing ATB-346 ^71^. Furthermore, Kodela et al. (2015) hypothesized that NOSH-aspirin may be especially promising with respect to improved renal safety profile, because both NO and H_2_S have been recognized as protective factors within renal system. Yet, further studies may confirm and support these conclusions. On the other hand, CO-releasing CORM-A1 applied to healthy mice notably increased renal blood flow, as compared to control group (RYAN et al., 2006). Noteworthy, CORM-A1 has been shown to relax renal interlobar arteries contracted with phenylephrine and this effect was dependent on guanylate cyclase activity together with the opening of potassium channels (RYAN et al., 2006). In spite of the essential role in physiological processes in kidneys, gaseous mediators released from various donors have been also demonstrated to possess therapeutic properties. In detail, Fujihara et al. (1998) reported that nitroflurbiprofen (HCT-1026) alleviated renal damage in a rat model of 5/6 kidney ablation, contrary to its parent drug, flurbiprofen. In addition, they also observed that HCT-1026 markedly ameliorated albuminuria, interstitial injury, retention of creatinine and hypertension in rats subjected to kidney ablation. In turn, Kim et al. (2020) have examined the therapeutic efficacy of CORM-3 against ischemia-reperfusion renal injury in rats. CORM-3 pretreatment significantly decreased the degree of kidney damage comparing to control group, which could be explained, at least in part, by diminished apoptotic activity evoked by this compound. Similarly, renoprotective potential of CORM-2 has been observed in a mice model of thermal injury (Kim et al., 2020). CO-releasing CORM-2 prevented granulocytes infiltration in renal tissue induced in response to thermal damage, as well as notably decreased NF-κB activation in kidney. To summarize, the numerous effects of gaseous mediators released from chemical donors, involving anti-inflammatory, anti-apoptotic and vasodilatory properties, could account for significant renoprotective action of these compounds, even offering a promising alternative in future therapies against various renal disorders.

## Conclusions and Further Perspectives

Overcoming the toxicity of NSAIDs, particularly the well-known GI adverse effects, still remains a target for further studies revolving around different preventive strategies including NSAIDs conjugated with gaseous mediators as one of the most interesting and promising groups. Animal studies as well as clinical trials concerning the efficacy and safety of these novel compounds have shown encouraging results, giving a solid background to prompt the further investigation. These novel NSAIDs or NSAIDs combined with CORMs were shown to modulate several molecular pathways (Table 1.).

**TABLE 1:** Alterations of selected molecular targets by donors of gaseous mediators NO- H_2_S- and CO -and NSAID prodrugs.

	Gaseous mediators-relesaing prodrug		
Molecular target	NO donors and NO-NSAIDs	H_2_S donors and H_2_S-NSAIDs	CO donors and NSAIDs
Nf-κB	Downregulation Bolla et al. (2006)	Downregulation Li et al. (2007)	Downregulation Ling et al. (2018)
Nrf-2	No reports	Upregulation of Nrf-2 expression in gastric mucosa, activation of Nrf-2/HMOX-1/CO pathway Magierowski et al. (2017), Corsello et al. (2018)	Activation of Nrf-2 signaling pathway in mouse macrophages Qin et al. (2015)
ERK/MAPK	Inhibition of MAPK pathway Rigas (2007)	Inhibition of MAPK pathway Guo et al. (2014)	Activation of MAPK pathway Ryter et al. (2018)
			Modulation of ERK/MAPK in T cells, supressing their profileration Głowacka et al. (2020)
COXs	COX-1 and COX-2 Downregulation Magierowski et al. (2015)	COX-2 downregulation Magierowski et al. (2017)	COX-2 downregulation Ling et al. (2018)
CO biosynthesis	No reports	Upregulatiom of Nrf-2/HMOX-1/CO pathway in gastric mucosa Magierowski et al. (2017)	Upregulation of HMOX-1 expression in gastric mucosa Magierowska et al. (2019)
H_2_S biosynthesis	Increase in CTH activation, upregulation of H_2_S biosynthesis in cultured aortic smooth muscle cells Zhao et al. (2001)	No reports	CBS inhibition, regulation of H_2_S biosynthesis Głowacka et al. (2020)
	No effect on H_2_S biosynthesis in endothelial cells Chen et al. (2014)		
NO biosynthesis	iNOS inhibition Rigas (2007)	Inhibition of LPS-induced iNOS overexpression Li et al. (2007), Campolo et al. (2014)	Endothelial NOS activation Ryter et al. (2018), Zuckerbraun et al. (2006)
gut microbiota	No reports	Facilitation of antimicrobial immunity, normalization of enteric microbiota profile Blackler et al. (2015)	Modulation of interplay between microbiota and mucosal immune system Głowacka et al. (2020)
		Restoration of microbiota biofilm Motta et al. (2015)	

Both NO-NSADs and H_2_S-NSAIDs but also CO donors applied in combination with NSAIDs have been proven to retain the anti-inflammatory activity of their parent drugs, however, with markedly reduced gastrotoxicity and also importantly, with reduced intestinal toxicity, both become subjects of rising clinical underestimated concern (Figure 2). The increasing awareness on the deleterious influence of NSAIDs on the intestinal homeostasis confirms the need for the development of novel strategies with diminished toxicity toward intestinal mucosa and quality of enteric microbiota. Importantly, novel gaseous mediator-releasing NSAIDs with greatly limited GI toxicity and more complex molecular activity than parent drugs could be considered to be repurposed into the GI disorders treatment, such as chronic inflammation and pre- and cancerous pathologies. Although some promising results have been achieved with respect to the gaseous mediator-releasing NSAIDs, further studies are still needed to address these interesting and emerging issues.

## References

[B1] Al-Swayeh O. A. Clifford R. H. Del Soldato P. Moore P. K. (2000). A Comparison of the Anti-inflammatory and Anti-nociceptive Activity of Nitroaspirin and Aspirin. Br. J. Pharmacol. 129 (2), 343–350. 10.1038/sj.bjp.0703064 10694241 PMC1571848

[B2] Ambrosini M. V. Mariucci G. Rambotti M. G. Tantucci M. Covarelli C. Angelis L. D. (2005). Ultrastructural Investigations on Protective Effects of NCX 4016 (Nitroaspirin) on Macrovascular Endothelium in Diabetic Wistar Rats. J. Submicrosc Cytol. Pathol. 37 (2), 205–213. http://www.ncbi.nlm.nih.gov/pubmed/16335593. 16335593

[B3] Bacchi S. Palumbo P. Sponta A. Coppolino M. F. (2012). Clinical Pharmacology of Non-steroidal Anti-inflammatory Drugs: A Review. Aiaamc 11 (1), 52–64. 10.2174/187152312803476255 22934743

[B4] Bakalarz D. Surmiak M. Yang X. Wójcik D. Korbut E. Śliwowski Z. (2021). Organic Carbon Monoxide Prodrug, BW-CO-111, in Protection against Chemically-Induced Gastric Mucosal Damage. Acta Pharmaceutica Sinica B 11 (2), 456–475. 10.1016/j.apsb.2020.08.005 33643824 PMC7893125

[B5] Bełtowski J. (2015). Hydrogen Sulfide in Pharmacology and Medicine – an Update. Pharmacol. Rep. 67 (3), 647–658. 10.1016/j.pharep.2015.01.005 25933982

[B6] Bindu S. Mazumder S. Bandyopadhyay U. (2020). Non-steroidal Anti-inflammatory Drugs (NSAIDs) and Organ Damage: A Current Perspective. Biochem. Pharmacol. 180, 114147. 10.1016/j.bcp.2020.114147 32653589 PMC7347500

[B7] Blackler R. Syer S. Bolla M. Ongini E. Wallace J. L. (2012). Gastrointestinal-Sparing Effects of Novel NSAIDs in Rats with Compromised Mucosal Defence. PLoS One 7 (4), e35196. 10.1371/journal.pone.0035196 22496907 PMC3322164

[B9] Blackler R. W. De Palma G. Manko A. Da Silva G. J. Flannigan K. L. Bercik P. (2015). Deciphering the Pathogenesis of NSAID Enteropathy Using Proton Pump Inhibitors and a Hydrogen Sulfide-Releasing NSAID. Am. J. Physiology-Gastrointestinal Liver Physiol. 308 (12), G994–G1003. 10.1152/ajpgi.00066.2015 25882612

[B8] Blackler R. W. Gemici B. Manko A. Wallace J. L. (2014). NSAID-gastroenteropathy: New Aspects of Pathogenesis and Prevention. Curr. Opin. Pharmacol. 19, 11–16. 10.1016/j.coph.2014.05.008 24929967

[B10] Boelsterli U. A. Redinbo M. R. Saitta K. S. (2013). Multiple NSAID-Induced Hits Injure the Small Intestine: Underlying Mechanisms and Novel Strategies. Toxicol. Sci. 131 (2), 654–667. 10.1093/toxsci/kfs310 23091168 PMC3551426

[B11] Bolla M. Momi S. Gresele P. Del Soldato P. (2006). Nitric Oxide-Donating Aspirin (NCX 4016): An Overview of its Pharmacological Properties and Clinical Perspectives. Eur. J. Clin. Pharmacol. 62 (1), 145–154. 10.1007/s00228-005-0026-6

[B12] Bonetti P. O. Lerman L. O. Lerman A. (2003). Endothelial Dysfunction. Atvb 23 (2), 168–175. 10.1161/01.ATV.0000051384.43104 12588755

[B13] Bruno G. Zaccari P. Rocco G. Scalese G. Panetta C. Porowska B. (2019). Proton Pump Inhibitors and Dysbiosis: Current Knowledge and Aspects to Be Clarified. Wjg 25 (22), 2706–2719. 10.3748/wjg.v25.i22.2706 31235994 PMC6580352

[B14] Brzozowska I. Targosz A. Sliwowski Z. Kwiecien S. Drozdowicz D. Pajdo R. (2004). Healing of Chronic Gastric Ulcers in Diabetic Rats Treated with Native Aspirin, Nitric Oxide (NO)-derivative of Aspirin and Cyclooxygenase (COX)-2 Inhibitor. J. Physiol. Pharmacol. 55 (4), 773–790. http://www.ncbi.nlm.nih.gov/pubmed/15613743. 15613743

[B15] Brzozowski T. Konturek P. C. Konturek S. J. Sliwowski Z. Drozdowicz D. Kwiecień S. (2000). Gastroprotective and Ulcer Healing Effects of Nitric Oxide-Releasing Non-steroidal Anti-inflammatory Drugs. Dig. Liver Dis. 32 (7), 583–594. 10.1016/S1590-8658(00)80840-3 11142556

[B16] Cabassi A. Tedeschi S. Perlini S. Verzicco I. Volpi R. Gonzi G. (2020). Non-steroidal Anti-inflammatory Drug Effects on Renal and Cardiovascular Function: from Physiology to Clinical Practice. Eur. J. Prev. Cardiolog 27 (8), 850–867. 10.1177/2047487319848105 31088130

[B17] Campolo M. Esposito E. Ahmad A. Di Paola R. Paterniti I. Cordaro M. (2014). Hydrogen Sulfide-Releasing Cyclooxygenase Inhibitor ATB-346 Enhances Motor Function and Reduces Cortical Lesion Volume Following Traumatic Brain Injury in Mice. J. Neuroinflammation 11 (1), 196. 10.1186/s12974-014-0196-1 25472548 PMC4265354

[B18] Caughey G. E. Roughead E. E. Vitry A. I. McDermott R. A. Shakib S. Gilbert A. L. (2010). Comorbidity in the Elderly with Diabetes: Identification of Areas of Potential Treatment Conflicts. Diabetes Res. Clin. Pract. 87 (3), 385–393. 10.1016/j.diabres.2009.10.019 19923032

[B20] Chattopadhyay M. Kodela R. Duvalsaint P. L. Kashfi K. (2016). Gastrointestinal Safety, Chemotherapeutic Potential, and Classic Pharmacological Profile of NOSH ‐naproxen ( AVT ‐219) a Dual NO‐ and H 2 S‐releasing Hybrid. Pharmacol. Res. Perspect. 4 (2), e00224. 10.1002/prp2.224 27069635 PMC4804313

[B19] Chattopadhyay M. Kodela R. Olson K. R. Kashfi K. (2012). NOSH-aspirin (NBS-1120), a Novel Nitric Oxide- and Hydrogen Sulfide-Releasing Hybrid Is a Potent Inhibitor of Colon Cancer Cell Growth In Vitro and in a Xenograft Mouse Model. Biochem. Biophysical Res. Commun. 419 (3), 523–528. 10.1016/j.bbrc.2012.02.051 22366248

[B21] Chattopadhyay M. Kodela R. Santiago G. Le T. T. C. Nath N. Kashfi K. (2020). NOSH-aspirin (NBS-1120) Inhibits Pancreatic Cancer Cell Growth in a Xenograft Mouse Model: Modulation of FoxM1, P53, NF-Κb, iNOS, Caspase-3 and ROS. Biochem. Pharmacol. 176, 113857. 10.1016/j.bcp.2020.113857 32061771 PMC7263941

[B22] Chen P.-H. Fu Y.-S. Wang Y.-M. Yang K.-H. Wang D. L. Huang B. (2014). Hydrogen Sulfide Increases Nitric Oxide Production and Subsequent S-Nitrosylation in Endothelial Cells. Scientific World J. 2014, 1–8. 10.1155/2014/480387 PMC4055124 24971375

[B23] Choi A. M. Alam J. (1996). Heme Oxygenase-1: Function, Regulation, and Implication of a Novel Stress-Inducible Protein in Oxidant-Induced Lung Injury. Am. J. Respir. Cel Mol Biol 15 (1), 9–19. 10.1165/ajrcmb.15.1.8679227 8679227

[B24] Cipriani S. Mencarelli A. Bruno A. Renga B. Distrutti E. Santucci L. (2013). Activation of the Bile Acid Receptor GPBAR1 Protects against Gastrointestinal Injury Caused by Non-steroidal Anti-inflammatory Drugs and Aspirin in Mice. Br. J. Pharmacol. 168 (1), 225–237. 10.1111/j.1476-5381.2012.02128.x 22881598 PMC3570017

[B25] Citi V. Piragine E. Testai L. Breschi M. C. Calderone V. Martelli A. (2018). The Role of Hydrogen Sulfide and H2S-Donors in Myocardial Protection against Ischemia/Reperfusion Injury. Cmc 25 (34), 4380–4401. 10.2174/0929867325666180212120504 29436990

[B26] Clark J. E. Naughton P. Shurey S. Green C. J. Johnson T. R. Mann B. E. (2003). Cardioprotective Actions by a Water-Soluble Carbon Monoxide-Releasing Molecule. Circ. Res. 93 (2), e2–8. 10.1161/01.RES.0000084381.86567.08 12842916

[B27] Conaghan P. G. (2012). A Turbulent Decade for NSAIDs: Update on Current Concepts of Classification, Epidemiology, Comparative Efficacy, and Toxicity. Rheumatol. Int. 32 (6), 1491–1502. 10.1007/s00296-011-2263-6 22193214 PMC3364420

[B28] Corsello T. Komaravelli N. Casola A. (2018). Role of Hydrogen Sulfide in NRF2- and Sirtuin-dependent Maintenance of Cellular Redox Balance. Antioxidants 7 (10), 129. 10.3390/antiox7100129 PMC6210431 30274149

[B29] de Araújo S. Oliveira A. P. Sousa F. B. M. Souza L. K. M. Pacheco G. Filgueiras M. C. (2018). AMPK Activation Promotes Gastroprotection through Mutual Interaction with the Gaseous Mediators H 2 S, NO, and CO. Nitric Oxide 78, 60–71. 10.1016/j.niox.2018.05.008 29857061

[B30] Del Soldato P. Sorrentino R. Pinto A. (1999). NO-aspirins: A Class of New Anti-inflammatory and Antithrombotic Agents. Trends Pharmacol. Sci. 20 (8), 319–323. 10.1016/S0165-6147(99)01353-X 10431210

[B31] Di Napoli M. Papa F. (2003). NCX-4016 NicOx. Curr. Opin. Investig. Drugs 4 (9), 1126–1139. http://www.ncbi.nlm.nih.gov/pubmed/14582459. 14582459

[B32] Dieppe P. A. Ebrahim S. Martin R. M. Jüni P. (2004). Lessons from the Withdrawal of Rofecoxib: ***. BMJ 329 (7471), 867–868. 10.1136/bmj.329.7471.867 15485938 PMC523096

[B33] Ekundi-Valentim E. Mesquita F. P. Santos K. T. de Paula M. A. Florenzano J. Zanoni C. I. (2013). A Comparative Study on the Anti-inflammatory Effects of Single Oral Doses of Naproxen and its Hydrogen Sulfide (H2S)-Releasing Derivative ATB-346 in Rats with Carrageenan-Induced Synovitis. Med. Gas Res. 3 (1), 24. 10.1186/2045-9912-3-24 24237604 PMC3843537

[B34] Elliott S. N. McKnight W. Cirino G. Wallace J. L. (1995). A Nitric Oxide-Releasing Nonsteroidal Anti-inflammatory Drug Accelerates Gastric Ulcer Healing in Rats. Gastroenterology 109 (2), 524–530. 10.1016/0016-5085(95)90341-0 7615202

[B35] Failli P. Vannacci A. Di Cesare Mannelli L. Motterlini R. Masini E. (2012). Relaxant Effect of a Water Soluble Carbon Monoxide-Releasing Molecule (CORM-3) on Spontaneously Hypertensive Rat Aortas. Cardiovasc. Drugs Ther. 26 (4), 285–292. 10.1007/s10557-012-6400-6 22766583

[B36] Fiorucci S. Antonelli E. Burgaud J.-L. Morelli A. (2001). Nitric Oxide???Releasing NSAIDs. Drug Saf. 24 (11), 801–811. 10.2165/00002018-200124110-00002 11665868

[B39] Fiorucci S. Antonelli E. Distrutti E. Rizzo G. Mencarelli A. Orlandi S. (2005). Inhibition of Hydrogen Sulfide Generation Contributes to Gastric Injury Caused by Anti-inflammatory Nonsteroidal Drugs. Gastroenterology 129 (4), 1210–1224. 10.1053/j.gastro.2005.07.060 16230075

[B38] Fiorucci S. Mencarelli A. Meneguzzi A. Lechi A. Renga B. del Soldato P. (2004). Co-Administration of Nitric Oxide-Aspirin (NCX-4016) and Aspirin Prevents Platelet and Monocyte Activation and Protects against Gastric Damage Induced by Aspirin in Humans. J. Am. Coll. Cardiol. 44 (3), 635–641. 10.1016/j.jacc.2004.03.079 15358033

[B37] Fiorucci S. Santucci L. Wallace J. L. Sardina M. Romano M. del Soldato P. (2003). Interaction of a Selective Cyclooxygenase-2 Inhibitor with Aspirin and NO-Releasing Aspirin in the Human Gastric Mucosa. Proc. Natl. Acad. Sci. 100 (19), 10937–10941. 10.1073/pnas.1933204100 12960371 PMC196906

[B40] Fonseca M. D. Cunha F. Q. Kashfi K. Cunha T. M. (2015). NOSH-aspirin (NBS-1120), a Dual Nitric Oxide and Hydrogen Sulfide-Releasing Hybrid, Reduces Inflammatory Pain. Pharmacol. Res. Perspect. 3 (3), e00133. 10.1002/prp2.133 26236481 PMC4492749

[B41] Foresti R. Hammad J. Clark J. E. Johnson T. R. Mann B. E. Friebe A. (2004). Vasoactive Properties of CORM-3, a Novel Water-Soluble Carbon Monoxide-Releasing Molecule. Br. J. Pharmacol. 142 (3), 453–460. 10.1038/sj.bjp.0705825 15148243 PMC1574979

[B42] Fournier J.-P. Sommet A. Bourrel R. Oustric S. Pathak A. Lapeyre-Mestre M. (2012). Non-steroidal Anti-inflammatory Drugs (NSAIDs) and Hypertension Treatment Intensification: a Population-Based Cohort Study. Eur. J. Clin. Pharmacol. 68 (11), 1533–1540. 10.1007/s00228-012-1283-9 22527348

[B43] Freitas A. Alves-Filho J. C. Secco D. D. Neto A. F. Ferreira S. H. Barja-Fidalgo C. (2006). Heme Oxygenase/carbon Monoxide-Biliverdin Pathway Down Regulates Neutrophil Rolling, Adhesion and Migration in Acute Inflammation. Br. J. Pharmacol. 149 (4), 345–354. 10.1038/sj.bjp.0706882 16953189 PMC1978436

[B44] Fujihara C. K. Malheiros D. M. A. C. Donato J. Poli A. De Nucci G. Zatz R. (1998). Nitroflurbiprofen, a New Nonsteroidal Anti-inflammatory, Ameliorates Structural Injury in the Remnant Kidney. Am. J. Physiology-Renal Physiol. 274 (3), F573–F579. 10.1152/ajprenal.1998.274.3.F573 9530274

[B46] Glowacka U. Magierowska K. Wojcik D. Hankus J., Szetela M. Cieszkowski J. (2021). Microbiome Profile and Molecular Pathways Alterations in Gastrointestinal Tract by Hydrogen Sulfide-Releasing Non-steroidal Anti-inflammatory Drug (ATB-352). Insight into Possible Safer Polypharmacy. Antioxid. Redox Signal. 2020, 8240. 10.1089/ars.2020.8240 33678013

[B47] Goldstein J. L. Eisen G. M. Lewis B. Gralnek I. M. Zlotnick S. Fort J. G. (2005). Video Capsule Endoscopy to Prospectively Assess Small Bowel Injury with Celecoxib, Naproxen Plus Omeprazole, and Placebo. Clin. Gastroenterol. Hepatol. 3 (2), 133–141. 10.1016/S1542-3565(04)00619-6 15704047

[B48] Graham D. Y. Opekun A. R. Willingham F. F. Qureshi W. A. (2005). Visible Small-Intestinal Mucosal Injury in Chronic NSAID Users. Clin. Gastroenterol. Hepatol. 3 (1), 55–59. 10.1016/S1542-3565(04)00603-2 15645405

[B50] Gresele P. Migliacci R. Procacci A. De Monte P. Bonizzoni E. (2007). Prevention by NCX 4016, a Nitric Oxide-Donating Aspirin, but Not by Aspirin, of the Acute Endothelial Dysfunction Induced by Exercise in Patients with Intermittent Claudication. Thromb. Haemost. 97 (03), 444–450. 10.1160/TH06-10-0555 17334512

[B49] Gresele P. Momi S. (2006). Pharmacologic Profile and Therapeutic Potential of NCX 4016, a Nitric Oxide-Releasing Aspirin, for Cardiovascular Disorders. Cardiovasc. Drug Rev. 24 (2), 148–168. 10.1111/j.1527-3466.2006.00148.x 16961726

[B51] Gugliandolo E. Fusco R. D’Amico R. Militi A. Oteri G. Wallace J. L. (2018). Anti-inflammatory Effect of ATB-352, a H2S −releasing Ketoprofen Derivative, on Lipopolysaccharide-Induced Periodontitis in Rats. Pharmacol. Res. 132, 220–231. 10.1016/j.phrs.2017.12.022 29287688

[B53] Guo C. Liang F. Shah Masood W. Yan X. (2014). Hydrogen Sulfide Protected Gastric Epithelial Cell from Ischemia/reperfusion Injury by Keap1 S-Sulfhydration, MAPK Dependent Anti-apoptosis and NF-Κb Dependent Anti-inflammation Pathway. Eur. J. Pharmacol. 725 (1), 70–78. 10.1016/j.ejphar.2014.01.009 24444438

[B52] Guo Y. Stein A. B. Wu W.-J. Tan W. Zhu X. Li Q.-H. (2004). Administration of a CO-releasing Molecule at the Time of Reperfusion Reduces Infarct Size In Vivo. Am. J. Physiology-Heart Circulatory Physiol. 286 (5), H1649–H1653. 10.1152/ajpheart.00971.2003 PMC3208268 14704226

[B45] Głowacka U. Brzozowski T. Magierowski M. (2020). Synergisms, Discrepancies and Interactions between Hydrogen Sulfide and Carbon Monoxide in the Gastrointestinal and Digestive System Physiology, Pathophysiology and Pharmacology. Biomolecules 10 (3), 445. 10.3390/biom10030445 PMC7175135 32183095

[B54] Harirforoosh S. Asghar W. Jamali F. (2014). Adverse Effects of Nonsteroidal Antiinflammatory Drugs: An Update of Gastrointestinal, Cardiovascular and Renal Complications. J. Pharm. Pharm. Sci. 16 (5), 821. 10.18433/J3VW2F 24393558

[B55] Higuchi K. Yoda Y. Amagase K. Kato S. Tokioka S. Murano M. (2009). Prevention of NSAID-Induced Small Intestinal Mucosal Injury: Prophylactic Potential of Lansoprazole. J. Clin. Biochem. Nutr. 45 (2), 125–130. 10.3164/jcbn.SR09-58 19794918 PMC2735622

[B56] Huang C. W. Moore P. K. (2015). H2S Synthesizing Enzymes: Biochemistry and Molecular Aspects. Handb Exp. Pharmacol. 230, 3–25. 10.1007/978-3-319-18144-8_1 26162827

[B57] Huang E. S. Strate L. L. Ho W. W. Lee S. S. Chan A. T. (2011). Long-Term Use of Aspirin and the Risk of Gastrointestinal Bleeding. Am. J. Med. 124 (5), 426–433. 10.1016/j.amjmed.2010.12.022 21531232 PMC3086018

[B58] Jasnos K. Magierowski M. Kwiecień S. Brzozowski T. (2014). Carbon Monoxide in Human Physiology - its Role in the Gastrointestinal Tract. Postepy Hig Med. Dosw 68, 101–109. 10.5604/17322693.1087527 24491901

[B59] Ji J. Xiang P. Li T. Lan L. Xu X. Lu G. (2017). NOSH-NBP, a Novel Nitric Oxide and Hydrogen Sulfide- Releasing Hybrid, Attenuates Ischemic Stroke-Induced Neuroinflammatory Injury by Modulating Microglia Polarization. Front. Cel. Neurosci. 11, 154. 10.3389/fncel.2017.00154 PMC5445131 28603491

[B60] Karlsson J. Pivodic A. Aguirre D. Schnitzer T. J. (2009). Efficacy, Safety, and Tolerability of the Cyclooxygenase-Inhibiting Nitric Oxide Donator Naproxcinod in Treating Osteoarthritis of the Hip or Knee. J. Rheumatol. 36 (6), 1290–1297. 10.3899/jrheum.081011 19411388

[B61] Kashfi K. Chattopadhyay M. Kodela R. (2015). NOSH-sulindac (AVT-18A) Is a Novel Nitric Oxide- and Hydrogen Sulfide-Releasing Hybrid that Is Gastrointestinal Safe and Has Potent Anti-inflammatory, Analgesic, Antipyretic, Anti-platelet, and Anti-cancer Properties. Redox Biol. 6, 287–296. 10.1016/j.redox.2015.08.012 26298203 PMC4556776

[B62] Kashfi K. (2015). Utility of Nitric Oxide and Hydrogen Sulfide-Releasing Chimeras as Anticancer Agents. Redox Biol. 5, 420. 10.1016/j.redox.2015.09.030 28162289

[B63] Kato S. Suzuki K. Ukawa H. Komoike Y. Takeuchi K. (2001). Low Gastric Toxicity of Nitric Oxide-Releasing Aspirin, NCX-4016, in Rats with Cirrhosis and Arthritis. Dig. Dis. Sci. 46 (8), 1690–1699. 10.1023/a:1010601520497 11508669

[B64] Kautz A. C. Kunz P. C. Janiak C. (2016). CO-releasing Molecule (CORM) Conjugate Systems. Dalton Trans. 45 (45), 18045–18063. 10.1039/C6DT03515A 27808304

[B65] Keeble J. Al-Swayeh O. A. Moore P. K. (2001). Vasorelaxant Effect of Nitric Oxide Releasing Steroidal and Nonsteroidal Anti-inflammatory Drugs. Br. J. Pharmacol. 133 (7), 1023–1028. 10.1038/sj.bjp.0704161 11487511 PMC1572867

[B67] Kim D. K. Shin S.-J. Lee J. Park S. Y. Kim Y. T. Choi H. Y. (2020). Carbon Monoxide-Releasing Molecule-3: Amelioration of Renal Ischemia Reperfusion Injury in a Rat Model. Investig. Clin. Urol. 61 (4), 441–451. 10.4111/icu.2020.61.4.441 PMC7329640 32666002

[B66] Kim H.-H. Choi S. (2018). Therapeutic Aspects of Carbon Monoxide in Cardiovascular Disease. Ijms 19 (8), 2381. 10.3390/ijms19082381 PMC6121498 30104479

[B68] Kimura H. (2014). Production and Physiological Effects of Hydrogen Sulfide. Antioxid. Redox Signaling 20 (5), 783–793. 10.1089/ars.2013.5309 PMC3910667 23581969

[B69] Kodela R. Chattopadhyay M. Kashfi K. (2012). NOSH-aspirin: A Novel Nitric Oxide-Hydrogen Sulfide-Releasing Hybrid: A New Class of Anti-inflammatory Pharmaceuticals. ACS Med. Chem. Lett. 3 (3), 257–262. 10.1021/ml300002m 22916316 PMC3423220

[B70] Kodela R. Chattopadhyay M. Kashfi K. (2013). Synthesis and Biological Activity of NOSH-Naproxen (AVT-219) and NOSH-Sulindac (AVT-18A) as Potent Anti-inflammatory Agents with Chemotherapeutic Potential. Med. Chem. Commun. 4 (11), 1472. 10.1039/c3md00185g PMC3835719 24273639

[B71] Kodela R. Chattopadhyay M. Velázquez-Martínez C. A. Kashfi K. (2015). NOSH-aspirin (NBS-1120), a Novel Nitric Oxide- and Hydrogen Sulfide-Releasing Hybrid Has Enhanced Chemo-Preventive Properties Compared to Aspirin, Is Gastrointestinal Safe with All the Classic Therapeutic Indications. Biochem. Pharmacol. 98 (4), 564–572. 10.1016/j.bcp.2015.09.014 26394025 PMC4656078

[B72] Konturek S. J. (1985). Gastric Cytoprotection. Scand. J. Gastroenterol. 20 (5), 543–553. 10.3109/00365528509089694 3895382

[B73] Kulkarni-Chitnis M. Njie-Mbye Y. F. Mitchell L. Robinson J. Whiteman M. Wood M. E. (2015). Inhibitory Action of Novel Hydrogen Sulfide Donors on Bovine Isolated Posterior Ciliary Arteries. Exp. Eye Res. 134, 73–79. 10.1016/j.exer.2015.04.001 25845295 PMC4426029

[B74] Lanas A. (2010). A Review of the Gastrointestinal Safety Data-Aa Gastroenterologist's Perspective. Rheumatology 49 (Suppl. 2), ii3–ii10. 10.1093/rheumatology/keq058 20407138 PMC2857792

[B75] Lee M. McGeer E. Kodela R. Kashfi K. McGeer P. L. (2013). NOSH-aspirin (NBS-1120), a Novel Nitric Oxide and Hydrogen Sulfide Releasing Hybrid, Attenuates Neuroinflammation Induced by Microglial and Astrocytic Activation: A New Candidate for Treatment of Neurodegenerative Disorders. Glia 61 (10), 1724–1734. 10.1002/glia.22553 23918470

[B76] Li L. Rossoni G. Sparatore A. Lee L. C. Del Soldato P. Moore P. K. (2007). Anti-inflammatory and Gastrointestinal Effects of a Novel Diclofenac Derivative. Free Radic. Biol. Med. 42 (5), 706–719. 10.1016/j.freeradbiomed.2006.12.011 17291994

[B77] Li L. Whiteman M. Guan Y. Y. Neo K. L. Cheng Y. Lee S. W. (2008). Characterization of a Novel, Water-Soluble Hydrogen Sulfide-Releasing Molecule (GYY4137). Circulation 117 (18), 2351–2360. 10.1161/CIRCULATIONAHA.107.753467 18443240

[B78] Ling K. Men F. Wang W.-C. Zhou Y.-Q. Zhang H.-W. Ye D.-W. (2018). Carbon Monoxide and its Controlled Release: Therapeutic Application, Detection, and Development of Carbon Monoxide Releasing Molecules (CORMs). J. Med. Chem. 61 (7), 2611–2635. 10.1021/acs.jmedchem.6b01153 28876065

[B79] Liu L. Cui J. Song C.-J. Bian J.-S. Sparatore A. Soldato P. D. (2012). H(2)S-Releasing Aspirin Protects against Aspirin-Induced Gastric Injury via Reducing Oxidative Stress. PLoS One 7 (9), e46301. 10.1371/journal.pone.0046301 23029468 PMC3460860

[B80] Liu Z. Han Y. Li L. Lu H. Meng G. Li X. (2013). The Hydrogen Sulfide Donor, GYY4137, Exhibits Anti-atherosclerotic Activity in High Fat Fed Apolipoprotein E−/−mice. Br. J. Pharmacol. 169 (8), 1795–1809. 10.1111/bph.12246 23713790 PMC3753836

[B81] LoGuidice A. Wallace B. D. Bendel L. Redinbo M. R. Boelsterli U. A. (2012). Pharmacologic Targeting of Bacterial β-Glucuronidase Alleviates Nonsteroidal Anti-inflammatory Drug-Induced Enteropathy in Mice. J. Pharmacol. Exp. Ther. 341 (2), 447–454. 10.1124/jpet.111.191122 22328575 PMC3336811

[B82] Lohmander L. S. (2004). A Randomised, Placebo Controlled, Comparative Trial of the Gastrointestinal Safety and Efficacy of AZD3582 versus Naproxen in Osteoarthritis. Ann. Rheum. Dis. 64 (3), 449–456. 10.1136/ard.2004.023572 15345500 PMC1755403

[B83] Lou L-X. Geng B. Du J-B. Tang C-S. (2008). Hydrogen Sulphide-Induced Hypothermia Attenuates Stress-Related Ulceration in Rats. Clin. Exp. Pharmacol. Physiol. 35 (2), 223–228. 10.1111/j.1440-1681.2007.04812.x 17941893

[B86] Magierowska K. Bakalarz D. Wójcik D. Chmura A. Hubalewska-Mazgaj M. Licholai S. (2019a). Time-dependent Course of Gastric Ulcer Healing and Molecular Markers Profile Modulated by Increased Gastric Mucosal Content of Carbon Monoxide Released from its Pharmacological Donor. Biochem. Pharmacol. 163, 71–83. 10.1016/j.bcp.2019.02.011 30753813

[B85] Magierowska K. Brzozowski T. Magierowski M. (2018). Emerging Role of Carbon Monoxide in Regulation of Cellular Pathways and in the Maintenance of Gastric Mucosal Integrity. Pharmacol. Res. 129, 56–64. 10.1016/j.phrs.2018.01.008 29360501

[B87] Magierowska K. Korbut E. Hubalewska-Mazgaj M. Surmiak M. Chmura A. Bakalarz D. (2019b). Oxidative Gastric Mucosal Damage Induced by Ischemia/reperfusion and the Mechanisms of its Prevention by Carbon Monoxide-Releasing Tricarbonyldichlororuthenium (II) Dimer. Free Radic. Biol. Med. 145, 198–208. 10.1016/j.freeradbiomed.2019.09.032 31568823

[B84] Magierowska K. Magierowski M. Surmiak M. Adamski J. Mazur-Bialy A. Pajdo R. (2016). The Protective Role of Carbon Monoxide (CO) Produced by Heme Oxygenases and Derived from the CO-Releasing Molecule CORM-2 in the Pathogenesis of Stress-Induced Gastric Lesions: Evidence for Non-involvement of Nitric Oxide (NO). Ijms 17 (4), 442. 10.3390/ijms17040442 27023525 PMC4848898

[B93] Magierowski M. Magierowska K. Surmiak M. Hubalewska-Mazgaj M., Kwiecien S. Wallace J. L. (2017b). The Effect of Hydrogen Sulfide-Releasing Naproxen (ATB-346) versus Naproxen on Formation of Stress-Induced Gastric Lesions, the Regulation of Systemic Inflammation, Hypoxia and Alterations in Gastric Microcirculation. J. Physiol. Pharmacol. 68 (5), 749–756. 29375050

[B95] Magierowski M. Hubalewska-Mazgaj M. Magierowska K. Wojcik D. Sliwowski Z. Kwiecien S. (2018b). Nitric Oxide, Afferent Sensory Nerves, and Antioxidative Enzymes in the Mechanism of Protection Mediated by Tricarbonyldichlororuthenium(II) Dimer and Sodium Hydrosulfide against Aspirin-Induced Gastric Damage. J. Gastroenterol. 53 (1), 52–63. 10.1007/s00535-017-1323-4 28238019

[B89] Magierowski M. Jasnos K. Kwiecien S. Drozdowicz D. Surmiak M. Strzalka M. (2015b). Endogenous Prostaglandins and Afferent Sensory Nerves in Gastroprotective Effect of Hydrogen Sulfide against Stress-Induced Gastric Lesions. PLoS One 10 (3), e0118972. 10.1371/journal.pone.0118972 25774496 PMC4361614

[B90] Magierowski M. Magierowska K. Hubalewska-Mazgaj M. Adamski J. Bakalarz D. Sliwowski Z. (2016a). Interaction between Endogenous Carbon Monoxide and Hydrogen Sulfide in the Mechanism of Gastroprotection against Acute Aspirin-Induced Gastric Damage. Pharmacol. Res. 114, 235–250. 10.1016/j.phrs.2016.11.001 27825819

[B92] Magierowski M. Magierowska K. Hubalewska-Mazgaj M. Sliwowski Z. Ginter G. Pajdo R. (2017a). Carbon Monoxide Released from its Pharmacological Donor, Tricarbonyldichlororuthenium (II) Dimer, Accelerates the Healing of Pre-existing Gastric Ulcers. Br. J. Pharmacol. 174 (20), 3654–3668. 10.1111/bph.13968 28768046 PMC5610153

[B94] Magierowski M. Magierowska K. Hubalewska-Mazgaj M. Surmiak M. Sliwowski Z. Wierdak M. (2018a). Cross-talk between Hydrogen Sulfide and Carbon Monoxide in the Mechanism of Experimental Gastric Ulcers Healing, Regulation of Gastric Blood Flow and Accompanying Inflammation. Biochem. Pharmacol. 149, 131–142. 10.1016/j.bcp.2017.11.020 29203367

[B88] Magierowski M. Magierowska K. Kwiecien S. Brzozowski T. (2015a). Gaseous Mediators Nitric Oxide and Hydrogen Sulfide in the Mechanism of Gastrointestinal Integrity, Protection and Ulcer Healing. Molecules 20 (5), 9099–9123. 10.3390/molecules20059099 25996214 PMC6272495

[B91] Magierowski M. Magierowska K. Szmyd J. Surmiak M. Sliwowski Z. Kwiecien S. (2016b). Hydrogen Sulfide and Carbon Monoxide Protect Gastric Mucosa Compromised by Mild Stress against Alendronate Injury. Dig. Dis. Sci. 61 (11), 3176–3189. 10.1007/s10620-016-4280-5 27541924 PMC5067292

[B96] Maiden L. Thjodleifsson B. Theodors A. Gonzalez J. Bjarnason I. (2005). A Quantitative Analysis of NSAID-Induced Small Bowel Pathology by Capsule Enteroscopy. Gastroenterology 128 (5), 1172–1178. 10.1053/j.gastro.2005.03.020 15887101

[B97] Mard S. A. Neisi N. Solgi G. Hassanpour M. Darbor M. Maleki M. (2012). Gastroprotective Effect of NaHS against Mucosal Lesions Induced by Ischemia-Reperfusion Injury in Rat. Dig. Dis. Sci. 57 (6), 1496–1503. 10.1007/s10620-012-2051-5 22271414

[B98] Matsui H. Shimokawa O. Kaneko T. Nagano Y. Rai K. Hyodo I. (2011). The Pathophysiology of Non-steroidal Anti-inflammatory Drug (NSAID)-induced Mucosal Injuries in Stomach and Small Intestine. J. Clin. Biochem. Nutr. 48 (2), 107–111. 10.3164/jcbn.10-79 21373261 PMC3045681

[B99] Mizoguchi H. Hase S. Tanaka A. Takeuchi K. (2001). Lack of Small Intestinal Ulcerogenecity of Nitric Oxide-Releasing Indomethacin, NCX-530, in Rats. Aliment. Pharmacol. Ther. 15 (2), 257–267. 10.1046/j.1365-2036.2001.00916.x 11148446

[B100] Momi S. Emerson M. Paul W. Leone M. Mezzasoma A. M. Del Soldato P. (2000). Prevention of Pulmonary Thromboembolism by NCX 4016, a Nitric Oxide-Releasing Aspirin. Eur. J. Pharmacol. 397 (1), 177–185. 10.1016/S0014-2999(00)00223-5 10844112

[B101] Mostafa D. K. El Azhary N. M. Nasra R. A. (2016). The Hydrogen Sulfide Releasing Compounds ATB-346 and Diallyl Trisulfide Attenuate Streptozotocin-Induced Cognitive Impairment, Neuroinflammation, and Oxidative Stress in Rats: Involvement of Asymmetric Dimethylarginine. Can. J. Physiol. Pharmacol. 94 (7), 699–708. 10.1139/cjpp-2015-0316 27088818

[B102] Motta J.-P. Flannigan K. L. Agbor T. A. Beatty J. K. Blackler R. W. Workentine M. L. (2015). Hydrogen Sulfide Protects from Colitis and Restores Intestinal Microbiota Biofilm and Mucus Production. Inflamm. Bowel Dis. 21 (5), 1006–1017. 10.1097/MIB.0000000000000345 25738373

[B104] Motterlini R. Clark J. E. Foresti R. Sarathchandra P. Mann B. E. Green C. J. (2002). Carbon Monoxide-Releasing Molecules. Circ. Res. 90 (2), E17–E24. 10.1161/hh0202.104530 11834719

[B103] Motterlini R. Otterbein L. E. (2010). The Therapeutic Potential of Carbon Monoxide. Nat. Rev. Drug Discov. 9 (9), 728–743. 10.1038/nrd3228 20811383

[B105] Mu K. Yu S. Kitts D. D. (2019). The Role of Nitric Oxide in Regulating Intestinal Redox Status and Intestinal Epithelial Cell Functionality. Ijms 20 (7), 1755. 10.3390/ijms20071755 PMC6479862 30970667

[B106] Mukherjee D. (2001). Risk of Cardiovascular Events Associated with Selective COX-2 Inhibitors. JAMA 286 (8), 954. 10.1001/jama.286.8.954 11509060

[B107] Muscará M. N. Lovren F. McKnight W. Dicay M. Soldato P. d. Triggle C. R. (2001). Vasorelaxant Effects of a Nitric Oxide-Releasing Aspirin Derivative in Normotensive and Hypertensive Rats. Br. J. Pharmacol. 133 (8), 1314–1322. 10.1038/sj.bjp.0704209 11498517 PMC1621160

[B109] Napoli C. Ackah E. de Nigris F. Del Soldato P. D'Armiento F. P. Crimi E. (2002). Chronic Treatment with Nitric Oxide-Releasing Aspirin Reduces Plasma Low-Density Lipoprotein Oxidation and Oxidative Stress, Arterial Oxidation-specific Epitopes, and Atherogenesis in Hypercholesterolemic Mice. Proc. Natl. Acad. Sci. 99 (19), 12467–12470. 10.1073/pnas.192244499 12209007 PMC129468

[B108] Napoli C. Cirino G. Del Soldato P. Sorrentino R. Sica V. Condorelli M. (2001). Effects of Nitric Oxide-Releasing Aspirin versus Aspirin on Restenosis in Hypercholesterolemic Mice. Proc. Natl. Acad. Sci. 98 (5), 2860–2864. 10.1073/pnas.041602898 11226331 PMC30230

[B110] Olas B. (2014). Carbon Monoxide Is Not Always a Poison Gas for Human Organism: Physiological and Pharmacological Features of CO. Chemico-Biological Interactions 222, 37–43. 10.1016/j.cbi.2014.08.005 25168849

[B111] Pereira-Leite C. Nunes C. Jamal S. K. Cuccovia I. M. Reis S. (2017). Nonsteroidal Anti-inflammatory Therapy: A Journey toward Safety. Med. Res. Rev. 37 (4), 802–859. 10.1002/med.21424 28005273

[B112] Pieper G. M. Siebeneich W. Olds C. L. Felix C. C. Soldato P. D. (2002). Vascular Protective Actions of a Nitric Oxide Aspirin Analog in Both In Vitro and In Vivo Models of Diabetes Mellitus. Free Radic. Biol. Med. 32 (11), 1143–1156. 10.1016/S0891-5849(02)00832-8 12031899

[B113] Qin S. Du R. Yin S. Liu X. Xu G. Cao W. (2015). Nrf2 Is Essential for the Anti-inflammatory Effect of Carbon Monoxide in LPS-Induced Inflammation. Inflamm. Res. 64 (7), 537–548. 10.1007/s00011-015-0834-9 26049867

[B114] Rigas B. (2007). Novel Agents for Cancer Prevention Based on Nitric Oxide. Biochem. Soc. Trans. 35 (5), 1364–1368. 10.1042/BST0351364 17956352

[B115] Robert A. Asano T. (1977). Resistance of Germfree Rats to Indomethacin-Induced Intestinal Lesions. Prostaglandins 14 (2), 333–341. 10.1016/0090-6980(77)90178-2 331401

[B116] Rolando B. Lazzarato L. Donnola M. Marini E. Joseph S. Morini G. (2013). Water-Soluble Nitric-Oxide-Releasing Acetylsalicylic Acid (ASA) Prodrugs. ChemMedChem 8 (7), 1199–1209. 10.1002/cmdc.201300105 23754790

[B117] Rossoni G. Berti M. Colonna V. D. Bernareggi M. Del Soldato P. Berti F. (2000). Myocardial Protection by the Nitroderivative of Aspirin, NCX 4016: In Vitro and In Vivo Experiments in the Rabbit. Ital. Heart J. 1 (2), 146–155. http://www.ncbi.nlm.nih.gov/pubmed/10730616. 10730616

[B118] Rossoni G. Manfredi B. Colonna V. D. Bernareggi M. Berti F. (2001). The Nitroderivative of Aspirin, NCX 4016, Reduces Infarct Size Caused by Myocardial Ischemia-Reperfusion in the Anesthetized Rat. J. Pharmacol. Exp. Ther. 297 (1), 380–387. http://www.ncbi.nlm.nih.gov/pubmed/11259566. 11259566

[B120] Rossoni G. Manfredi B. Tazzari V. Sparatore A. Trivulzio S. Del Soldato P. (2010). Activity of a New Hydrogen Sulfide-Releasing Aspirin (ACS14) on Pathological Cardiovascular Alterations Induced by Glutathione Depletion in Rats. Eur. J. Pharmacol. 648 (1-3), 139–145. 10.1016/j.ejphar.2010.08.039 20826133

[B119] Rossoni G. Sparatore A. Tazzari V. Manfredi B. Soldato P. D. Berti F. (2008). The Hydrogen Sulphide-Releasing Derivative of Diclofenac Protects against Ischaemia-Reperfusion Injury in the Isolated Rabbit Heart. Br. J. Pharmacol. 153 (1), 100–109. 10.1038/sj.bjp.0707540 17965734 PMC2199380

[B121] Ryan M. Jernigan N. Drummond H. Mclemorejr G. Rimoldi J. Poreddy S. (2006). Renal Vascular Responses to CORM-A1 in the Mouse. Pharmacol. Res. 54 (1), 24–29. 10.1016/j.phrs.2006.01.012 16524742

[B122] Ryter S. W. Ma K. C. Choi A. M. K. (2018). Carbon Monoxide in Lung Cell Physiology and Disease. Am. J. Physiology-Cell Physiol. 314 (2), C211–C227. 10.1152/ajpcell.00022.2017 PMC5866434 29118026

[B123] Scheiman J. M. Yeomans N. D. Talley N. J. Vakil N. Chan F. K. L. Tulassay Z. (2006). Prevention of Ulcers by Esomeprazole in At-Risk Patients Using Non-selective NSAIDs and COX-2 Inhibitors. Am. J. Gastroenterol. 101 (4), 701–710. 10.1111/j.1572-0241.2006.00499.x 16494585

[B124] Schnitzer T. J. Kivitz A. J. Lipetz R. S. Sanders N. Hee A. (2005). Comparison of the COX-Inhibiting Nitric Oxide Donator AZD3582 and Rofecoxib in Treating the Signs and Symptoms of Osteoarthritis of the Knee. Arthritis Rheum. 53 (6), 827–837. 10.1002/art.21586 16342089

[B125] Schroeder B. O. Wu Z. Nuding S. Groscurth S. Marcinowski M. Beisner J. (2011). Reduction of Disulphide Bonds Unmasks Potent Antimicrobial Activity of Human β-defensin 1. Nature 469 (7330), 419–423. 10.1038/nature09674 21248850

[B126] Shi Y. Vanhoutte P. M. (2017). Macro- and Microvascular Endothelial Dysfunction in Diabetes. J. Diabetes 9 (5), 434–449. 10.1111/1753-0407.12521 28044409

[B127] Shindo K. Machida M. Fukumura M. Koide K. Yamazaki R. (1998). Omeprazole Induces Altered Bile Acid Metabolism. Gut 42 (2), 266–271. 10.1136/gut.42.2.266 9536953 PMC1727000

[B128] Srinivasan A. De Cruz P. (2017). Review Article: a Practical Approach to the Clinical Management of NSAID Enteropathy. Scand. J. Gastroenterol. 52 (9), 1–7. 10.1080/00365521.2017.1335769 28587496

[B129] Stanek A. Gadowska-Cicha A. Gawron K. Wielkoszynski T. Adamek B. Cieslar G. (2008). Role of Nitric Oxide in Physiology and Pathology of the Gastrointestinal Tract. Mrmc 8 (14), 1549–1560. 10.2174/138955708786786462 19075811

[B130] Sulaieva O. Wallace J. L. (2015). Gaseous Mediator-Based Anti-inflammatory Drugs. Curr. Opin. Pharmacol. 25, 1–6. 10.1016/j.coph.2015.08.005 26319186

[B131] Sun H.-Z. Zheng S. Lu K. Hou F.-T. Bi J.-X. Liu X.-L. (2017). Hydrogen Sulfide Attenuates Gastric Mucosal Injury Induced by Restraint Water-Immersion Stressviaactivation of KATPchannel and NF-Κb Dependent Pathway. Wjg 23 (1), 87. 10.3748/wjg.v23.i1.87 28104983 PMC5221289

[B132] Syer S. D. Blackler R. W. Martin R. de Palma G. Rossi L. Verdu E. (2015). NSAID Enteropathy and Bacteria: a Complicated Relationship. J. Gastroenterol. 50 (4), 387–393. 10.1007/s00535-014-1032-1 25572030

[B133] Tai F. W. D. McAlindon M. E. (2018). NSAIDs and the Small Bowel. Curr. Opin. Gastroenterol. 34 (3), 175–182. 10.1097/MOG.0000000000000427 29438118

[B134] Takagi T. Naito Y. Uchiyama K. Mizuhima K. Suzuki T. Horie R. (2016). Carbon Monoxide Promotes Gastric Wound Healing in Mice via the Protein Kinase C Pathway. Free Radic. Res. 50 (10), 1098–1105. 10.1080/10715762.2016.1189546 27170088

[B135] Takahashi T. Shimizu H. Morimatsu H. Maeshima K. Inoue K. Akagi R. (2009). Heme Oxygenase-1 Is an Essential Cytoprotective Component in Oxidative Tissue Injury Induced by Hemorrhagic Shock. J. Clin. Biochem. Nutr. 44 (1), 28–40. 10.3164/jcbn.08-210-HO 19177185 PMC2613496

[B136] Takeuchi K. Ukawa H. Konaka A. Kitamura M. Sugawa Y. (1998). Effect of Nitric Oxide-Releasing Aspirin Derivative on Gastric Functional and Ulcerogenic Responses in Rats: Comparison with Plain Aspirin. J. Pharmacol. Exp. Ther. 286 (1), 115–121. http://www.ncbi.nlm.nih.gov/pubmed/9655849. 9655849

[B137] Takeuchi K. Mizoguchi H. Araki H. Komoike Y. Suzuki K. (2001). Lack of Gastric Toxicity of Nitric Oxide-Releasing Indomethacin, NCX-530, in Experimental Animals. Dig. Dis. Sci. 46 (8), 1805–1818. 10.1023/a:1010638528675 11508687

[B138] Topol E. J. (2005). Arthritis Medicines and Cardiovascular Events-"House of Coxibs". JAMA 293 (3), 366. 10.1001/jama.293.3.366 15623849

[B139] Van Dingenen J. Pieters L. Vral A. Lefebvre R. A. (2019). The H2S-Releasing Naproxen Derivative ATB-346 and the Slow-Release H2S Donor GYY4137 Reduce Intestinal Inflammation and Restore Transit in Postoperative Ileus. Front. Pharmacol. 10, 10–116. 10.3389/fphar.2019.00116 30842737 PMC6391894

[B140] Vannini F. Kodela R. Chattopadhyay M. Kashfi K. (2015a). NOSH-aspirin Inhibits Colon *Cancer* Cell Growth: Effects of Positional Isomerism. Redox Biol. 5, 421. 10.1016/j.redox.2015.09.033 28162291

[B141] Vannini F. MacKessack-Leitch A. C. Eschbach E. K. Chattopadhyay M. Kodela R. Kashfi K. (2015b). Synthesis and Anti-cancer Potential of the Positional Isomers of NOSH-Aspirin (NBS-1120) a Dual Nitric Oxide and Hydrogen Sulfide Releasing Hybrid. Bioorg. Med. Chem. Lett. 25 (20), 4677–4682. 10.1016/j.bmcl.2015.08.023 26323873 PMC4592841

[B142] Varadi J. Lekli I. Juhasz B. Bacskay I. Szabo G. Gesztelyi R. (2007). Beneficial Effects of Carbon Monoxide-Releasing Molecules on Post-ischemic Myocardial Recovery. Life Sci. 80 (17), 1619–1626. 10.1016/j.lfs.2007.01.047 17321552

[B143] Varga Z. Sabzwariali S. r. a. Vargova V. (2017). Cardiovascular Risk of Nonsteroidal Anti-inflammatory Drugs: An Under-recognized Public Health Issue. Cureus 9 (4), e1144. 10.7759/cureus.1144 28491485 PMC5422108

[B147] Wallace J. L. Elliott S. N. Del Soldato P. McKnight W. Sannicolo F. Cirino G. (1997). Gastrointestinal-sparing Anti-inflammatory Drugs: The Development of Nitric Oxide-Releasing NSAIDs. Drug Dev. Res. 42 (3-4), 144–149. 10.1002/(SICI)1098-2299(199711/12)42:3/4<144::AID-DDR5>3.0.CO;2-Q

[B149] Wallace J. L. Caliendo G. Santagada V. Cirino G. Fiorucci S. (2007). Gastrointestinal Safety and Anti-inflammatory Effects of a Hydrogen Sulfide-Releasing Diclofenac Derivative in the Rat. Gastroenterology 132 (1), 261–271. 10.1053/j.gastro.2006.11.042 17241876

[B151] Wallace J. L. Ianaro A. de Nucci G. (2017). Gaseous Mediators in Gastrointestinal Mucosal Defense and Injury. Dig. Dis. Sci. 62 (9), 2223–2230. 10.1007/s10620-017-4681-0 28733867

[B146] Wallace J. L. McKnight W. Del Soldato P. Baydoun A. R. Cirino G. (1995). Anti-thrombotic Effects of a Nitric Oxide-Releasing, Gastric-Sparing Aspirin Derivative. J. Clin. Invest. 96 (6), 2711–2718. 10.1172/JCI118338 8675638 PMC185978

[B157] Wallace J. L. (2013). Mechanisms, Prevention and Clinical Implications of Nonsteroidal Anti-inflammatory Drug-Enteropathy. Wjg 19 (12), 1861. 10.3748/wjg.v19.i12.1861 23569332 PMC3613102

[B148] Wallace J. L. Muscara M. N. McKnight W. Dicay M. Del Soldato P. Cirino G. (1999). *In Vivo* Antithrombotic Effects of a Nitric Oxide-Releasing Aspirin Derivative, NCX-4016. Thromb. Res. 93 (1), 43–50. 10.1016/S0049-3848(98)00134-0 10065898

[B144] Wallace J. L. Muscará M. N. (2001). Selective Cyclo-Oxygenase-2 Inhibitors: Cardiovascular and Gastrointestinal Toxicity. Dig. Liver Dis. 33, S21–S28. 10.1016/S1590-8658(01)80155-9 11827359

[B153] Wallace J. L. Nagy P. Feener T. D. Allain T. Ditrói T. Vaughan D. J. (2020). A Proof‐of‐concept, Phase 2 Clinical Trial of the Gastrointestinal Safety of a Hydrogen Sulfide‐releasing Anti‐inflammatory Drug. Br. J. Pharmacol. 177 (4), 769–777. 10.1111/bph.14641 30834513 PMC7024706

[B156] Wallace J. L. (2012). NSAID Gastropathy and Enteropathy: Distinct Pathogenesis Likely Necessitates Distinct Prevention Strategies. Br. J. Pharmacol. 165 (1), 67–74. 10.1111/j.1476-5381.2011.01509.x 21627632 PMC3252967

[B155] Wallace J. L. (2010). Physiological and Pathophysiological Roles of Hydrogen Sulfide in the Gastrointestinal Tract. Antioxid. Redox Signaling 12 (9), 1125–1133. 10.1089/ars.2009.2900 19769457

[B154] Wallace J. L. (2008). Prostaglandins, NSAIDs, and Gastric Mucosal Protection: Why Doesn't the Stomach Digest Itself?. Physiol. Rev. 88 (4), 1547–1565. 10.1152/physrev.00004.2008 18923189

[B150] Wallace J. L. Syer S. Denou E. de Palma G. Vong L. McKnight W. (2011). Proton Pump Inhibitors Exacerbate NSAID-Induced Small Intestinal Injury by Inducing Dysbiosis. Gastroenterology 141 (4), 1314–1322. 10.1053/j.gastro.2011.06.075 21745447

[B152] Wallace J. L. Vaughan D. Dicay M. MacNaughton W. K. de Nucci G. (2018). Hydrogen Sulfide-Releasing Therapeutics: Translation to the Clinic. Antioxid. Redox Signaling 28 (16), 1533–1540. 10.1089/ars.2017.7068 28388861

[B145] Wallace J. L. Wang R. (2015). Hydrogen Sulfide-Based Therapeutics: Exploiting a Unique but Ubiquitous Gasotransmitter. Nat. Rev. Drug Discov. 14 (5), 329–345. 10.1038/nrd4433 25849904

[B158] Warner T. D. Nylander S. Whatling C. (2011). Anti-platelet Therapy: Cyclo-Oxygenase Inhibition and the Use of Aspirin with Particular Regard to Dual Anti-platelet Therapy. Br. J. Clin. Pharmacol. 72 (4), 619–633. 10.1111/j.1365-2125.2011.03943.x 21320154 PMC3195738

[B159] Watanabe T. Tanigawa T. Nadatani Y. Nagami Y. Sugimori S. Okazaki H. (2013). Risk Factors for Severe Nonsteroidal Anti-inflammatory Drug-Induced Small Intestinal Damage. Dig. Liver Dis. 45 (5), 390–395. 10.1016/j.dld.2012.12.005 23333664

[B160] Whelton A. (1999). Nephrotoxicity of Nonsteroidal Anti-inflammatory Drugs: Physiologic Foundations and Clinical Implications. Am. J. Med. 106 (5), 13S–24S. 10.1016/S0002-9343(99)00113-8 10390124

[B161] Wu L. Wang R. (2005). Carbon Monoxide: Endogenous Production, Physiological Functions, and Pharmacological Applications. Pharmacol. Rev. 57 (4), 585–630. 10.1124/pr.57.4.3 16382109

[B162] Zanardo R. C. O. Brancaleone V. Distrutti E. Fiorucci S. Cirino G. Wallace J. L. (2006). Hydrogen Sulfide Is an Endogenous Modulator of Leukocyte‐mediated Inflammation. FASEB j. 20 (12), 2118–2120. 10.1096/fj.06-6270fje 16912151

[B163] Zanellato I. Bonarrigo I. Ravera M. Gabano E. Gust R. Osella D. (2013). The Hexacarbonyldicobalt Derivative of Aspirin Acts as a CO-releasing NSAID on Malignant Mesothelioma Cells. Metallomics 5 (12), 1604. 10.1039/c3mt00117b 24057048

[B164] Zhao W. Zhang J. Lu Y. Wang R. (2001). The Vasorelaxant Effect of H2S as a Novel Endogenous Gaseous KATP Channel Opener. EMBO J. 20 (21), 6008–6016. 10.1093/emboj/20.21.6008 11689441 PMC125693

[B165] Zuckerbraun B. S. Chin B. Y. Wegiel B. Billiar T. R. Czsimadia E. Rao J. (2006). Carbon Monoxide Reverses Established Pulmonary Hypertension. J. Exp. Med. 203 (9), 2109–2119. 10.1084/jem.20052267 16908624 PMC2118401

